# Fine‐Tuning the Microstructure and Photophysical Characteristics of Fluorescent Conjugated Copolymers Using Photoalignment and Liquid‐Crystal Ordering

**DOI:** 10.1002/advs.202407117

**Published:** 2024-08-29

**Authors:** Yuping Shi, Katharina Landfester, Stephen M. Morris

**Affiliations:** ^1^ Max Planck Institute for Polymer Research Ackermannweg 10 55128 Mainz Germany; ^2^ Department of Engineering Science University of Oxford Parks Road Oxford OX1 3PJ UK

**Keywords:** biomimetic materials, chain alignment, förster resonance energy transfer, liquid crystals, organic donor–acceptor system, polarized emission, semiconducting conjugated polymers

## Abstract

Replicating the microstructural basis and the near 100% excitation energy transfer efficiency in naturally occurring light‐harvesting complexes (LHCs) remains challenging in synthetic energy‐harvesting devices. Biological photosynthesis regulates active ensembles of light‐absorbing and funneling chlorophylls in proteins in response to fluctuating sunlight. Here, use of long‐range liquid crystal (LC) ordering to tailor chain orientation and packing structure in liquid crystalline conjugated polymer (LCCP) layers for bio‐mimicry of certain structural basis and light‐harvesting properties of LHCs is reported. It is found that long‐range orientational ordering in an LC phase of poly(9,9‐dioctylfluorene‐co‐benzothiadiazole) (F8BT) copolymer stabilizes a small fraction of randomly‐oriented F8BT nanocrystals dispersed in an amorphous matrix of F8BT chains, resembling a self‐doped host–guest system whereby excitation energy funneling and photoluminescence quantum efficiencies are enhanced significantly by triggering 3D donor‐to‐acceptor Förster resonance energy transfer (FRET) and dominant intrachain emission in the nano‐crystal acceptor. Further, photoalignment of nematic F8BT layers is combined with LC orientational ordering to fabricate large‐area‐extended monodomains exhibiting >60% crystallinity and ≈20 nm‐long interchain packing order. Remarkably, these monodomains demonstrate strong linearly polarized emission, whilst also promoting a new band‐edge absorption species and an extra emissive interchain excited state as compared to the non‐aligned films.

## Graphical abstract

A combination of high‐quality photoalignment and liquid‐crystal (LC) ordering is demonstrated to create long‐range structural ordering and anisotropic coupling into otherwise amorphous conjugated polymer deposits, resembling a remarkably efficient self‐doped host–guest system for biomimicking natural light‐harvesting complexes. Photoaligned (PA) polymer films exhibit large‐area‐extended monodomain texture with near‐unity order parameter, ≈100% intrachain photoluminescence (PL) quantum efficiency, and linearly polarized emission.

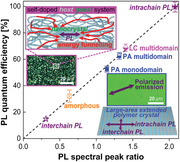

## 1 Introduction

Nature is a constant and unparalleled source of inspiration for microstructural design and many‐body coupling optimization. Higher plants and many algae are able to make efficient use of physical–chemical tuning of protein‐templated pigment chromophores within a light‐harvesting complex (LHC) in order to fully support and spontaneously regulate their photosynthetic activities.^[^ ^1^ ^]^ Absorbed photon energy is spatially funneled at ultra‐low energy losses among LHCs and ultimately arrives at an energetically expensive reaction center for charge separation and chemical transformations.^[^ ^2^, ^3^ ^]^ Consecutive FRET or even quantum coherent energy transfer between these chemically similar yet differently‐sized LHCs is unique in terms of their ability to enable near‐unity efficiencies of excitation energy funneling from multiple minor chromophoric LHCs (LH2) to a structurally identical but larger‐sized major acceptor complex (LH1).^[^ ^4^, ^5^ ^]^ These multichromophoric LH1 and LH2 complexes in ubiquitous photosystems have evolved naturally to take an elegant ring‐shaped packing microstructure with precisely templated chromophoric molecules by a protein matrix,^[^ ^1^, ^2^, ^3^, ^4^, ^5^ ^]^ which could serve as a useful guide for the design and manufacture of man‐made light‐harvesting and energy‐conversion devices. In order to mimic the structural basis and coherent excitation energy funneling in natural LHCs, it is viable to create biomimetic material hybrids with an optimal number of minor amorphous chromophoric units that peripheralize and couple effectively with a larger yet lower‐energy core as an acceptor; in this way, the randomly‐oriented peripheral chromophores can facilitate the maximization of photon absorption for all light polarization states;^[^ ^6^ ^]^ while an appropriate energy landscape in an engineered donor–acceptor molecular network gives rise to optimal excitation energy transfer mediated by ultralow‐loss 3D FRET.^[^ ^7^, ^8^ ^]^ This demands fine‐tuning of the physical size, molecular arrangement, and electronic coupling within and between the donor and acceptor constitutes.

In contrast to the chemical homology naturally selected in LHCs,^[^ ^9^ ^]^ the use of chemically distinct species has been employed in a variety of organic semiconducting devices in order to imitate the excitation energy funneling and biophotocatalysis in LHCs. However, engineering chemical structures itself typically causes non‐negligible overpotentials and energy losses^[^ ^10^, ^11^, ^12^, ^13^, ^14^, ^15^ ^]^ owing to the presence of intrinsic energy‐gap mismatch and heterogeneous interfaces;^[^ ^16^, ^17^, ^18^, ^19^ ^]^ however, this could be mitigated or remedied by utilizing the tunability of physical structures of the same chemical species. To do so, developing techniques that allow us to engineer the physical structures of a high‐performing polymeric semiconductor (with spatial repetition of an identical chemical structure along its backbone) into an efficient “self‐doped” biomimetic donor–acceptor system would be a promising endeavor in our quest for ultrahigh light‐harvesting or energy transfer efficiencies. This calls for the realization of precise spatial definition of chain conformation, interchain packing, and macroscopic domain texture in a cost effective device‐relevant layer of the same conjugated polymer.^[^ ^20^, ^21^, ^22^, ^23^, ^24^, ^25^ ^]^ In this regard, solution‐processable conjugated copolymers can be best‐in‐class multi‐chromophoric systems, especially when the effective π‐conjugation is coherently interrupted due to twisted or bent backbones.^[^ ^6^, ^26^ ^]^ This site‐dependent structural feature tends to permit a straight chain segment of a polymer backbone to act as either a chromophoric or a fluorophoric element depending on energy levels that are sensitive to the degree of chain extension and interchain packing structure. Therefore, a properly engineered polymeric semiconductor system can resemble a favorable host–guest system imitating the almost lossless energy transfer and structural regulation of photosynthetic organisms. For example, self‐doping a polymer layer by tailoring its intrachain conjugation, interchain packing, and electronic/vibrational coupling would facilitate selective activation and more efficient transport of the competing excited interchain and interchain states.^[^ ^6^ ^]^ Within this framework, molecular organization by virtue of the inherent long‐range orientational order present in a liquid crystal (LC) mesophase of light‐emitting liquid crystalline conjugated polymers (LCCPs)^[^ ^27^, ^28^ ^]^ is highly desirable.

Here, we report the fabrication and systematic tuning of nematic polydomain and monodomain films of thermotropic poly(9,9‐dioctylfluorene‐co‐benzothiadiazole) (F8BT). Its order parameter (S) ranges from 0 to ≈100%, and the relative weighting between its intrachain and interchain light emission is modulated through the long‐range orientational ordering in the nematic mesophase. A highly efficient host–guest system is thus generated from this novel self‐doping method, whereby both the excitation energy funneling efficiency and photoluminescence quantum efficiency (PLQE) are remarkably enhanced. The relative fraction of (disordered) amorphous chromophoric host in the self‐doped F8BT nematic polydomain film is also engineered to boost PLQE up to >70%, relative to a PLQE of ≈30% in the spin‐coated non‐LC analog. The improved light‐harvesting and energy‐conversion efficiencies in these bi‐phase nematic polydomain films arise potentially from the synergy between the promotion of intrachain emission in F8BT nanocrystals as acceptor and the deactivation of energetic traps existing in a major faction of the amorphous matrix of F8BT chains, mediated by a biomimetic FRET process from the amorphous chromophoric host to a small fraction (<5%) of randomly oriented F8BT nanocrystals as the guest inclusion. However, further increases in overall crystallinity to ≈60–70% and order parameter to *S* ≈1 as a result of high‐quality photoalignment of chain orientation in the high‐temperature nematic phase of F8BT, and then, freezing the structural order into a nematic monodomain glass film by a quenching step, is found to produce only an intermediate PLQE of ≈50% as a result of notably elongated interchain packing coherence and emergence of an additionally allowed emissive interchain species whose PLQE is projected to be <15%. The benefits and huge potential of engineering the relative ratio of F8BT nanocrystal acceptor in LC‐organized F8BT polydomain glass films are showcased via tailoring the spatially averaged size of nematic polydomains by control over the film thickness. Thus, this study exemplifies the feasibility of utilizing a self‐doped LCCP system to complement previous investigations of the structure–property relationship in non‐oriented F8BT and related copolymers, whilst also reporting progress toward biomimicry of the unique structural basis and energy transfer properties of LHCs.

## 2 Results and Discussion

### 2.1 Materials and Film Preparation

**Figure 1:**
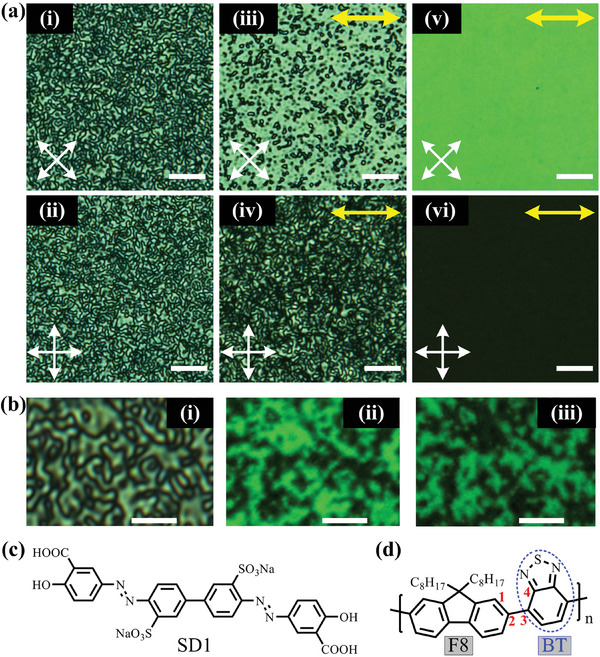
Observation of distinct LC textures in three comparative F8BT nematic glass films. a) Polarizing optical micrographs (POMs) demonstrating a‐i–iv) polydomain LC texture in both the nonaligned (Film I) (a‐i, ii) and partially‐aligned (Film III) (a‐iii, iv) nematic films and a‐v, vi) monodomain texture in the fully‐aligned nematic film (Film II), viewed between crossed polarizers (labeled by the two crossed white arrows). The photoaligned chain‐orientation direction is indicated by the double‐headed yellow arrows in (iii–vi), and all scale bars are 20 µm. b) A POM image at a higher magnification showing b‐i) typical Schlieren texture in a nonaligned nematic Film I and b‐ii, iii) corresponding polarized confocal fluorescence images recorded when F8BT light emission was collected with the analyzer aligned to two orthogonal (i.e., horizontal and vertical) polarization directions. The fluorescence was photoexcited with randomly polarized light at 450 nm; the scale bars in (b) are 5 µm. c, d) Chemical structures of SD1 (c) and F8BT (d). The blue ellipse in (d) encloses the BT moiety and the four carbons labeled by the red numbers resemble a dihedral (i.e., intermolecular torsion) angle linking the F8 and BT units through a single carbon─carbon bond around which the F8 and BT moieties have a certain degree of freedom to rotate relative to each other.

The pristine LCCP samples were prepared by spin coating F8BT on a pre‐cleaned Spectrosil substrate to form films with thickness ranging from 40 to 480 nm. Prior to the deposition of an F8BT layer, a continuous (discontinuous) ultrathin layer of our photoalignment material, namely hydrophilic sulphonic azo‐benzene dye (SD1), was pre‐coated directly onto a substrate using a 0.5 mg mL^−1^ (0.1 mg mL^−1^) solution, and then, aligned using linearly polarized ultraviolet (UV) illumination at *λ* = 365 nm from a light‐emitting diode (LED). The polarized UV illumination oriented the long molecular axis of SD1 molecules in the plane of the substrate along a direction that was orthogonal to the polarization of the UV light source.^[^ ^27^, ^28^, ^29^ ^]^ Subsequently, thermotropic LC‐alignment or photoalignment of the polymer chain orientation in an F8BT film or an SD1/F8BT bilayer was implemented by heating F8BT to its nematic mesophase before rapidly quenching to room temperature into a nematic glass (solid) film. This quenching step prevented polymer crystallization and thereby locked in the self‐assembled nematic director configuration, that is, a polydomain LC texture in a heated single layer of F8BT that had been directly coated on a substrate with no alignment layer (termed as nonaligned [NA] F8BT nematic glass film; labelled as Film I), and a monodomain texture for an F8BT film coated on a UV‐aligned SD1 photoalignment layer (termed as fully aligned [FA] F8BT nematic films; labeled as Film II). These highly oriented nematic monodomains were made possible by the long‐range orientational ordering in the nematic phase via molecular interaction between the UV‐aligned continuous SD1 layer and F8BT polymer chains at the interface, which was then communicated through the bulk of the LCCP layer by chain–chain interactions and π–π stacking. In addition, partially‐aligned (PA) F8BT nematic glass films (labeled as Film III) were fabricated by quenching a heated overlying F8BT film that had been coated on a UV‐aligned discontinuous SD1 layer resulting from spin‐coating on a quartz substrate with a low concentration (herein 0.1 mg mL^−1^) SD1 solution. Finally, the extensively studied spin‐coated (SC) F8BT films were fabricated on a quartz substrate but without going through the nematic phase for use as control samples (denoted as Film 0) for microstructural and photophysical characterization. All F8BT films studied in this work were 160 nm in thickness unless stated otherwise. Refer to **Figure** **1** for the chemical structures of F8BT and SD1, and to Figure S2, Supporting Information for a schematic illustration of the four types of comparative F8BT films studied within this work.

### 2.2 Microstructural Characterization and Chain‐Packing Model

Polarized optical micrographs (POMs) were recorded to visualize the LC texture formed in the F8BT films when each sample was placed between a crossed polarizer and analyzer pair. An F8BT nematic domain showed a dark (or bright) state when the local chain orientation (which defines the optic axis) was parallel (or at 45°) to the transmission axis of the polarizer or analyzer, whereas the spin‐coated non‐LC Film 0 appeared completely dark because of no birefringence effect. A typical Schlieren polydomain texture (i and ii in Figure 1a) was observed in the mesophase self‐assembled F8BT glass film (Film I). The direction of fluorophoric F8BT backbones in each nematic micro‐domain was observed to be well‐oriented by the LC long‐range orientational ordering, evidenced by the highly polarized fluorescence in Figure 1b; the local chain orientations of luminescent F8BT among different nematic micro‐domains; however, were still randomly distributed, as confirmed by the varying fluorescence intensity across the polydomain texture. High‐quality chain alignment in a large‐area‐extended nematic monodomain is highlighted in terms of the uniform bright‐state and dark‐state POMs (v and vi in Figure 1a) of the fully‐aligned F8BT nematic glass film (Film II).

**Figure 2:**
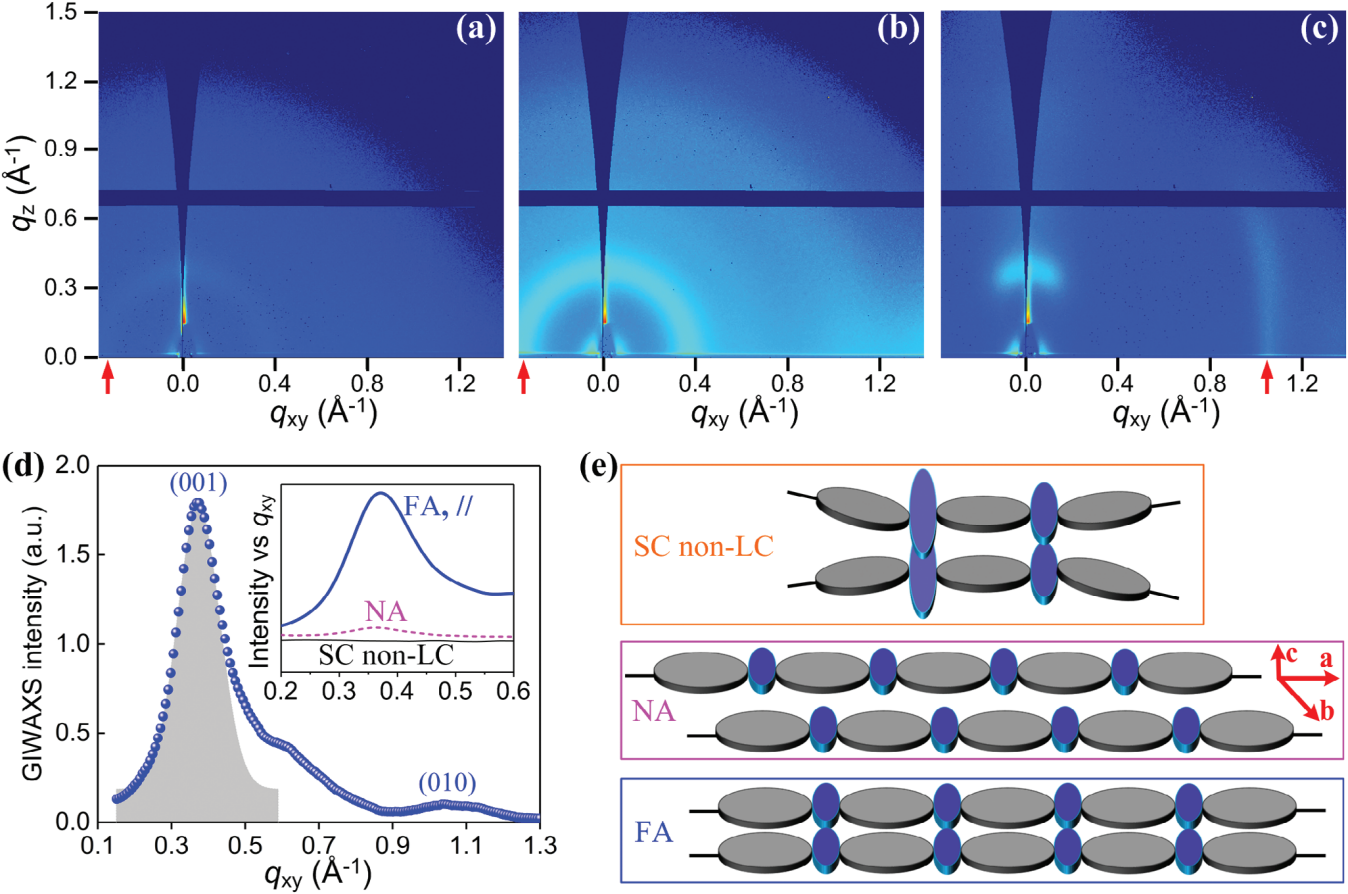
2D GIWAXS images of a) a nonaligned F8BT nematic polydomain Film I and b,c) a fully‐aligned F8BT nematic monodomain Film II, recorded with the incident X‐ray beam propagating in the SD1‐aligned film along a direction parallel (b) or perpendicular (c) to the chain alignment direction. The vertical red arrows on the bottom of (a–c) mark the *q_xy_* position of the characteristic scattering peaks. d) In‐plane linecut GIWAXS profile (blue dot) of the fully‐aligned Film II after subtracting a second order baseline, recorded with the parallel (//) configuration. The shaded region under the as‐shown Gaussian peak fitting curve in (d) represents the area of the (001) peak used to estimate the degree of overall crystallinity; the inset plots the raw scattering profile (offset for clarity) of the fully‐aligned (FA from the // configuration) and nonaligned (NA) F8BT nematic glass films and of a spin‐coated (SC) F8BT film (Film 0). e) Schematic illustration of the proposed model for polymer chain‐conformation and interchain packing structures in the three types of F8BT films. In (e), the grey and blue ellipses denote F8 and BT moiety along an F8BT backbone, where the side‐chains and backbone kinks are omitted for clarity; *a*, *b*, and *c* axes in the middle panel are shown along the backbone, interchain, and out‐of‐plane direction, respectively.

Grazing‐incidence wide‐angle X‐ray scattering (GIWAXS) measurement (**Figure** **2**a–d; Figure S3, Supporting Information) was carried out to clarify the microstructure and interchain packing in the F8BT films resulting from the different fabrication processes. The 2‐D GIWAXS patterns for both the nonaligned polydomain Film I and the fully‐aligned monodomain Film II are shown in Figure 2a–c, all of which were presented after subtracting the GIWAXS data of the spin‐coated reference Film 0 as the background in order to uncover the effect of LC‐alignment and photoalignment on long‐range (i.e., small *q*) structural ordering. Overall, the GIWAXS patterns show a big difference in crystallinity level, interchain packing, and structural anisotropy in different F8BT films. For the spin‐coated non‐LC Film 0, we see no clear indication of long‐range ordering, evidenced by the absence of a clear characteristic diffraction peak. The nonaligned nematic polydomain Film I exhibits a faint smearing ring (see Figure 2a and the insert in Figure 2d), which can be taken as a result of randomly distributed chain orientations in a small proportion of localized crystalline regions; this also points to the inclusion of ordered “polymer nanocrystals” in an amorphous phase matrix of disordered F8BT chains.^[^ ^30^ ^]^

The noticeably higher diffraction signals and asymmetric GIWAXS patterns from the fully‐aligned nematic monodomain Film II strongly suggest a substantial increase in the degree of polymer crystallinity and structural anisotropy. Figure 2b–d shows a rather strong diffraction ring recorded with a parallel X‐ray incident configuration, alongside two different diffraction arcs from a perpendicular measurement configuration—a smeared (100) arc and a sharper (010) arc. The appearance of the short (100) arc suggests the formation of highly oriented backbones in the extended nematic monodomain and that the relative rotation of F8 and BT moieties is restricted with both chemical units being aligned largely parallel to the plane of the substrate. The sharper (010) arc at *q_xy_* = 1.055 Å^−1^ gives an interchain repeat distance of 6.0 Å, which is a rather small periodicity when taking into consideration the length of the long alkyl side chains; this interchain repeat distance also corresponds to a condensed interchain packing pattern with prevalent BT‐to‐BT close contacts between adjacent chains.

The *q* values of the characteristic diffraction peaks and extracted *d*‐spacing and coherence lengths for the GIWAXS results are listed in **Table** **1**. Altogether, these diffraction results substantiate the feasibility of utilizing mesophase self‐assembly to induce polymer nanocrystal dispersions in the nonaligned F8BT nematic Film I, where an extended chain conformation with a slightly longer (by 3%) monomer length along the backbone direction and larger but 3D isotropic coherence lengths (141.7–149.6 Å) is locked‐in. The highly oriented nematic monodomain exhibits highly anisotropic coherence lengths, that is, 120.8, 205.1, and 101.0 Å along the backbone, interchain, and film thickness directions, respectively. The degree of polymer crystallinity over the representative photoaligned F8BT nematic monodomain is estimated to be ≈60–73%, quantified in terms of the area ratio of the (001) and (010) peaks in the total diffraction intensity when we took the shaded region under the Gaussian peak fitting curve in Figure 1d as the area of the (001) diffraction peak. The fraction of F8BT nanocrystals in the nonaligned polydomain Film I has an estimate of 4–5% calculated from the relative intensity of the faint (100) ring in the insert of Figure 2d.

**Table 1:** The extracted *d*‐spacing distances and coherence lengths from the 2D GIWAXS images recorded from the nonaligned and fully‐aligned F8BT nematic films.

F8BT sample	Direction	Peak	*q* [Å^−1^]	*d*‐spacing [Å]	Coherence length [Å]
Nonaligned	In‐plane	(100)	0.365	17.3	141.7
Aligned, //	In‐plane	(001)	0.374	16.8	120.8
Aligned, ⊥	In‐plane	(010)	1.055	6.0	205.1
Nonaligned	Out‐of‐plane	(100)	0.394	15.9	149.6
Aligned, //	Out‐of‐plane	(100)	0.385	16.3	98.4
Aligned, ⊥	Out‐of‐plane	(100)	0.387	16.2	103.5

The spontaneous mesophase structural self‐reorganization in the nonaligned polydomain Film I involves the stabilization of a minor fraction of the highly‐ordered polymer nano‐crystalline nuclei (which have a lower energy bandgap) within a pool of disordered nematic F8BT chains that have a chain length distribution defined by polydispersity. We expect these highly localized crystallization events to initialize through LC self‐assembly and associated chain extension of the most rigid (or straight) chain segments on the longest F8BT backbones, which would continue to selectively incorporate the next most extended chains in the mesophase into the already crystallized cores.^[^ ^30^ ^]^ For the fully‐ and partially‐aligned nematic F8BT films, a similar LC‐alignment process may proceed via a gradual expansion of the polymer nanocrystals at the expense of domain boundaries and re‐orientation of the newly incorporated F8BT chains to a director direction defined by the UV‐aligned SD1 commanding layer.

On the basis of all of our GIWAXS and POM results, an F8BT packing model has been proposed for different F8BT films (schematically illustrated in Figure 2e) to explain their different structural features and photophysical properties (vide infra). For the spin‐coated non‐LC film (Film 0), BT moieties have relatively random orientations but a high degree of intermolecular torsion with respect to the F8 unit (inherited from the significant structural disorders in an F8BT solution) and remain close to each other in neighboring chains to minimize steric hindrance. Such highly twisted molecular arrangement agrees with a dominance of the amorphous phase and significant BT‐to‐BT close contacts identified in Film 0. On the contrary, LC long‐range chain ordering induces polymer nanocrystals in the nonaligned nematic polydomain Film I through the re‐structuring of F8BT chains into a more energetically favorable configuration via adopting more planar chain conformations and dramatic suppression of intermolecular distortions; in this case, BT moieties in one F8BT chain would prefer to occupy positions adjacent to the F8 location of a neighboring chain (thus, recoiling the so‐called “alternating structure”) due to the repulsive force between BT‐to‐BT inter‐chain dipoles.^[^ ^31^ ^]^ Analogously, the fully‐aligned monodomain Film II can favor a more extended and rigid chain‐conformation but, in this case, the highly oriented F8BT chains translate relative to each other by half a repeat unit along the backbone direction; this aids the promotion of a higher number density of close interchain BT‐to‐BT contacts, particularly those under the templating effect imposed by the UV‐oriented (and probably cross‐linked) SD1 molecules.

### 2.3 Photophysical Characterization

The inherent mesophase long‐range orientational ordering of light‐emitting LCCPs creates a self‐doped host–guest system in which the disordered amorphous (higher‐energy) host acts as the chromophoric donor, while the self‐assembled polymer nanocrystals become the fluorophoric or acceptor constituent.^[^ ^26^, ^27^ ^]^ While the amorphous host and polymer nanocrystals both contribute to the dielectric and optical absorption behaviors of a bi‐phase film, the self‐doped polymer nanocrystals would dictate the emission properties by facilitating nonradiative host‐to‐guest FRET and deactivating possible traps in the major amorphous host. As a result, tailoring the effective π‐conjugation, the spatial distribution, and the relative weight of the host component would offer an intriguing means to fine‐tune the optical, electrical, and photophysical properties of light‐emitting LCCPs, which seems feasible for mimicking the ability of light‐harvesting antennas to regulate the number ratio of active minor‐ and larger‐sized LHCs in response to fluctuating sunlight.^[^ ^1^, ^5^ ^]^ In the case of LCCP F8BT, the lowest unoccupied molecular orbital (LUMO) localizes on the BT moieties, and the highest occupied molecular orbital (HOMO) delocalizes over the entire backbone.^[^ ^31^, ^32^, ^33^, ^34^ ^]^ Therefore, the absorption, migration, and decay of absorbed photon energy in the LC‐assembled and photoaligned F8BT glass films are fundamentally governed by the intrinsic competition among the structurally allowed intrachain and interchain excited states.

**Figure 3:**
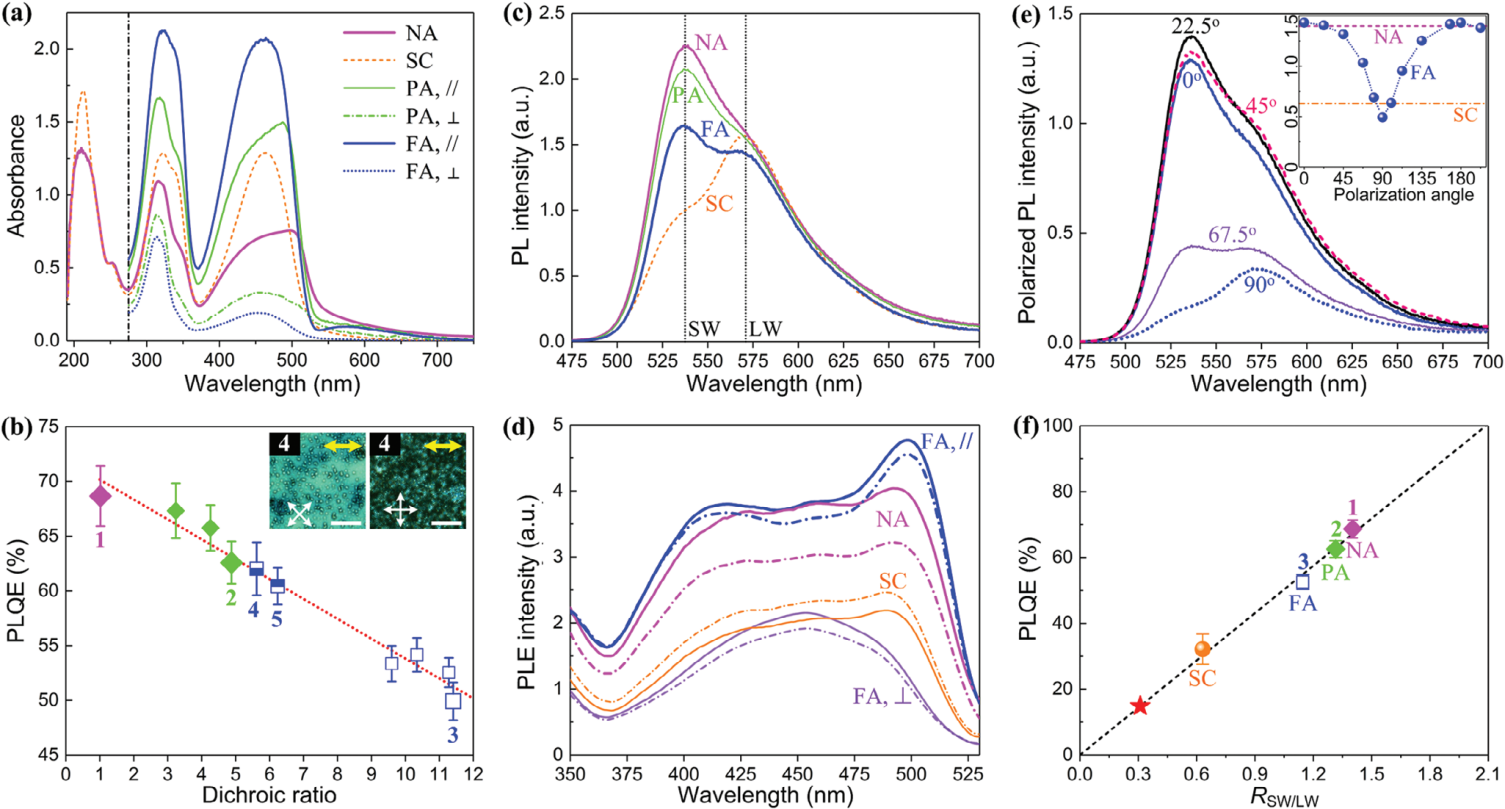
Photophysical characterization of the spin‐coated (SC) non‐LC F8BT film and of the nonaligned (NA), partially‐aligned (PA), and fully‐aligned (FA) F8BT nematic glass films. All parallel (//) and perpendicular (⊥) polarized spectra in the PA and FA films were recorded by aligning the excitation polarization parallel and perpendicular to the chain alignment direction, respectively. a) Optical absorption spectra of the four comparative F8BT films; the polarized spectra were cut off at 275 nm below which the transmission of the polarizer dropped rapidly, whereas the non‐polarized spectra for both the SC reference film and the NA nematic film were extended to 190 nm. b) Plot of PLQE in different nematic glass films of F8BT versus optical dichroic ratio determined at 460 nm. The data presented by the four blue opened boxes came from the fully‐aligned nematic monodomain films, and the fully‐filled diamond and half‐filled box data came from the nematic polydomain films of either NA (#1) and PA (#2) type, or a fully‐aligned but thinner F8BT film (e.g., sample #4: 40 nm thick, and sample #5: 80 nm thick). The inserts in (b) display the bright‐state (left) and dark‐state (right) POMs of sample #4, with scale bars being 20 µm. c) Non‐polarized PL spectra and d) polarized‐excited and nonpolarized‐collected PL excitation (PLE) spectra of various F8BT films. The two vertical dotted lines in (c) mark the identified shorter‐wavelength (SW) and longer‐wavelength (LW) PL peaks at ≈536 nm and 570 nm, which were assigned to the center of two vibronic optical transition bands. In (d), all solid curves were recorded for emission at 536 nm, while the dotted curves were for emission at 570 nm. e) Polarized PL spectra of the fully‐aligned nematic monodomain Film II probed under randomly polarized excitation light source and when emission was collected with polarization aligned at the as‐labelled polarization angles (*θ*) relative to the chain alignment direction. The insert in (e) plots the PL intensity ratio of the monodomain Film II at the SW (536 nm) to LW (570 nm) vibronic peak, *R*_SW/LW_, as a function of the emission collection polarization angle *θ* alongside the *R*_SW/LW_ of the nonaligned polydomain Film I (pink dashed line) and the spin‐coated Film 0 (orange dash‐dotted line). f) PLQE versus *R*_SW/LW_ for the four comparative F8BT films, where the red star denotes an extrapolated PLQE for so‐called “pure amorphous‐phase F8BT.” For the spin‐coated reference film, we also included the data measured from its annealed films (heating at 80 °C and 100 °C for 30 mins in N_2_ atmosphere), which led to a slightly larger variance of data in (f). The dotted and dashed line in (b,f) is a guide for a linear and a proportional change trend, respectively. All PLQE and PL spectra were measured using the same randomly polarized light source at 450 nm.

Polarized optical absorption spectra are collected to elaborate the electronic states and optical transitions in the comparative F8BT films. The absorption spectrum from the nonaligned F8BT nematic polydomain Film I (**Figure** **3**a) demonstrates a broadening of the lowest‐lying absorption band toward longer wavelengths, indicating the emergence of a new lower‐energy absorption species corresponding to F8BT nanocrystals therein. The peak wavelength of this first‐allowed optical transition band is blue‐shifted from 498 nm in the nonaligned polydomain Film I to 486 nm in the partially‐aligned nematic polydomain Film III. This is a signature of the continuing incorporation of higher‐energy chains during the growth of polymer nanocrystals; the peak wavelength further reduces to 465 nm in the non‐LC Film 0 and to 460 nm in the fully‐aligned F8BT nematic monodomain Film II as a result of the spatial average effect of a range of chain lengths. The absorption strength of the extended nematic monodomain is seen to be augmented relative to both the nonaligned polydomain Film I and the non‐LC Film 0 of the same thickness because the photoaligned backbones could remain largely parallel to the substrate plane and the chains in F8BT nanocrystals; while amorphous matrices are randomly oriented, resulting in the attenuation of oscillation strength when measurements are taken along the thickness of the film. Domain boundary regions separating nematic polydomains are expected to affect attenuated absorption strength similarly. The long‐wavelength spectral tail of the nonaligned polydomain Film I is ascribed to light scattering due to the structural heterogeneities associated with the randomly‐oriented nanocrystals.

The polarized absorption spectra of the fully‐aligned Film II emphasize high‐quality chain orientation and uniform monodomain texture; this manifests in the form of an ultrahigh optical dichroic ratio (i.e., the absorbance ratio between parallel [A*_//_*] and perpendicular [A*_⊥_*] polarized spectra to the chain alignment direction) of 11.4 at the peak wavelength of the first absorption band and the equivalent order parameter of *S* ≈0.98.^[^ ^27^ ^]^ The combined effects of highly oriented backbones in the plane of the substrate and the elimination of domain boundaries in the fully‐aligned monodomain Film II are essential for creating such an exceptional level of structural ordering and anisotropy in photophysical properties. The texture uniformity in Film II is also evident from the long wavelength interference fringes seen in its *A_//_* spectrum.

Overall, the absorption spectra in Figure 3a are consistent with the identified microstructures and proposed F8BT packing structures. The absorption peak intensity ratio between the first (375–500 nm wavelength) and second (275–375 nm) optical transition band increases from ≈2/3 in the nonaligned nematic polydomain Film I to levels close to 1 in both the spin‐coated non‐LC Film 0 and the fully‐aligned nematic monodomain Film II. This observation infers that F8BT chains in both Film 0 and Film II are largely restricted in the plane of the substrate, whereas F8BT chains in the polymer nanocrystals and amorphous matrix in Film I are randomly oriented in the 3D space. Observations of an increase in the first and second transition peak intensity ratio with chain alignment quality would help to rationalize the following points: i) spatial excitation energy transfer and concomitant first optical transition band associated with a charge‐transfer character involving BT units through intrachain π‐conjugation or BT‐to‐BT interchain coupling and ii) both the intrachain and interchain interactions are potentially enhanced by the high‐quality polymer photoalignment and BT‐to‐BT interchain alignment. In addition, the peak strength of the third transition band (centered around 200 nm, originating from the optical oscillation of benzene rings^[^ ^31^, ^34^ ^]^) in the spin‐coated Film 0 is determined from Figure 3a to be 1.3 times that of the nonaligned nematic glass film, arising from a random 3D distribution of F8BT chain orientations in the polymer nanocrystals and amorphous matrix. The second absorption band consisting of two transition constituents centered at 315 and 340 nm manifests distinct vibronic structures in the four types of F8BT films, in agreement with their different π–π stacking of F8BT chains.^[^ ^34^ ^]^ In particular, the lowest areal fraction of the 340 nm vibronic sideband in the second absorption band can be related to an alternating packing structure as proposed for the nonaligned F8BT nematic Film I, where fewer BT‐to‐BT close contacts and a larger extent of interchain misalignment are present; the disordered chain‐conformation and interchain packing in the non‐LC Film 0 has a moderate relative weight of the said 340 nm vibronic sideband due to significantly more BT‐to‐BT close contacts between adjacent chains and enhancement in interchain interactions. Among all measured F8BT films, the fully‐aligned monodomain Film II exhibits the highest fraction of 340 nm vibronic sidebands in the *A_//_* spectrum and also the lowest relative weight in the *A_⊥_* spectrum, which are in accordance with the observation of improved crystallinity and interchain coherent length.

In Figure 3b, we illustrate an unusual linear dependence of the PLQE in the F8BT nematic glass films on the chain‐alignment quality (parametrized as the optical dichroic ratio). A maximum enhanced PLQE of 68.8% ± 2.6% is shown for the nonaligned nematic polydomain Film I in comparison with the PLQE of 31.7% ± 5% in the spin‐coated non‐LC Film 0. While the PLQE values for the spin‐coated layers of non‐LC F8BT remain consistent with previous reports,^[^ ^31^, ^35^ ^]^ it is noteworthy that the difference between a PLQE of 71.6% ± 3.5% determined from F8BT solution and our maximized PLQE in the nonaligned polydomain *Film I* is within the measurement error. The nematic polydomains in either the nonaligned Film I or in the partially‐aligned Film III display a PLQE remarkably larger than that of the fully‐aligned monodomain Film II (≈50%). This linear PLQE change trend is further supported by the non‐polarized PL spectra shown in Figure 3c and polarized PL excitation (PLE) spectra in Figure 3d, in the form of a good agreement between the PL/PLE intensities and PLQE value. Although all of the measured F8BT films show a well‐resolved vibronic PL peak at ≈536 nm and another longer‐wavelength vibronic optical transition band centered at ≈570 nm, the relative PL peak intensity ratio of the short‐wavelength to long‐wavelength vibronic PL peak, *R*_SW/LW_, differs in different films (see also Figure S4a, Supporting Information for their normalized PL spectra): the non‐polarized PL spectra from both the nonaligned polydomain Film I and the partially‐aligned polydomain Film III are dominated by the first allowed (0–0) vibronic transition band, while the fully‐aligned monodomain Film II shows a relatively low *R*_SW/LW_ value which is still greater than one; on the other hand, the spin‐coated Film 0 exhibits the largest fraction of the longer‐wavelength vibronic transition in the PL spectrum.

These distinct *R*_SW/LW_ results and PL peak intensities among all F8BT films are in line with their PLE spectra acquired for emission at 536 and 570 nm (Figure 3d). Except Film 0, all F8BT films give a stronger PLE intensity for the 0–0 vibronic PL peak than emission at 570 nm. Considering Film 0 has a nearly amorphous feature and no long‐range exciton migration pathways, its highly twisted chain conformations would favor relatively lower‐energy optical transitions. Entering the LC mesophase in all the LC‐assembled F8BT glass films, on the other hand, improves PLQE by promoting the photo‐excitation of higher‐energy (0–0) vibronic emission because of enhanced intra/inter‐chain coherence lengths and mitigated chain entanglements. For instance, an ensuing increase in effective π‐conjugation length delocalizes excitonic wavefunction and allows access to extra emissive intrachain and interchain species of LC‐oriented F8BT chains, evidenced by the appearance of a new lower‐energy PLE sub‐band (peaking at a longer wavelength) that we have shown for the nonaligned and fully‐aligned nematic glass films (see Figure 3d). In contrast to Film 0 and nonaligned nematic polydomain Film I whose PLE spectra are almost independent of the excitation polarization, the fully‐aligned nematic monodomain Film II shows strongly polarization‐dependent PLE properties in correspondence with the anisotropic structural order and improved intra/inter‐chain coherence lengths. Under parallel polarized photo‐excitation, the PLE spectra in the extended nematic monodomains recorded for emission at 536 and 570 nm give stronger emission across the narrow longer‐wavelength sub‐band and also a PLE plateau extended to shorter wavelengths relative to the PLE spectra of the nonaligned nematic polydomain and non‐LC films. The clearly extended PLE spectrum points out the feasibility of high‐quality and long‐range polymer alignment for mitigating energetic disorder and activating band‐edge and intra‐band states.^[^ ^36^ ^]^ A decrease in the range of ordered interchain packing such as in F8BT nanocrystals allows to promote only the said additional band‐edge states, probably due to increasing energetic disorder along the interchain direction prior to the occurrence of light emission. When pumping with perpendicular polarized light, the two PLE spectra of the fully‐aligned Film II become relatively featureless, narrowed in wavelength range and lower in intensity because the reduced optical absorption may be insufficient to activate the sub‐band and band‐edge states. Spatial averaging of the parallel and perpendicular polarized PLE spectra in Film II lowers the total PLE intensity and the PLQE to a level that is less than those of the nonaligned nematic polydomain Film I.

The anisotropic PLE spectra and intermediate PLQE of the fully‐aligned F8BT nematic monodomain Film II commensurate with the polarized PL spectra shown in Figure 3e. The PL intensity of the 0–0 vibronic transition band is augmented when emission is collected with a polarization direction at a smaller angle (i.e., *θ*_Em_ = 0–45°) to the chain alignment direction and is maximized at *θ*_Em_ = 22.5°. There also exists a gradual shift in the *θ*_Em_‐dependence of relative PL peak intensity between two vibronic PL bands centered at 536 and 570 nm (see the insert in Figure 3e), with the lower‐lying vibronic transition band maximizing its fraction at *θ*_Em_ = 90°. Also shown in Figure 3e is the intensity of the 45°‐polarized PL spectrum exceeding the 22.5°‐polarized PL spectrum at wavelengths of >553 nm; we attribute this phenomenon to a competition between the gradually reduced intensity of the linearly polarized intrachain emission as *θ*_Em_ increases from 22.5° to 90°, and there is a simultaneous increase in intensity of an emissive excited species with an interchain character.^[^ ^27^ ^]^ Accordingly, the different *θ*_Em_‐dependence of the two orthogonal PL components is accompanied by a more complicated angular variation of *R*_SW/LW_ than a simple cos^2^(*θ*_Em_) relation expected for a single polarized emissive species. We propose that the discrepancy of the fluorescent excited intrachain and interchain species can be correlated with the possible prohibition of the 0–0 vibronic transition of interchain emission, in comparison with emissive intrachain species that are shown to allow 0–0, 0–1, and 0–2 vibronic transitions peaking at ≈536, 553, and 570 nm, respectively. Altogether, our vibronic PL spectral features, along with the polarization‐dependent PLE properties in the fully‐aligned monodomain Film II, encourage further assignment of so‐called *J*‐type and *H*‐type aggregation state^[^ ^25^ ^]^ to the emissive excited intrachain and interchain state, respectively. Note that *J*‐aggregation can trigger the occurrence of superradiation; and therefore high PLQE values while typical *H*‐aggregates usually lead to a far lower overall PLQE.^[^ ^37^ ^]^

Additional polarized PL spectra of the fully‐aligned F8BT nematic monodomain film, detected using four combinations of excitation (*θ*_Ext_) and emission collection (*θ*_Em_) polarization angle relative to F8BT chain alignment direction, are provided in Figures S4b and S5, Supporting Information to underline ultrahigh PL polarization anisotropies: a total PL intensity ratio of 28.6 and a PL peak intensity ratio of 40.4 at 536 nm between PL spectrum detected at *θ*_Em_ = 0°, *θ*_Ext_ = 0° and *θ*_Em_ = 90°, *θ*_Ext_ = 90°. These exceptional PL polarization anisotropies showcase the unique advantages of making use of the polarization degree of freedom for selective photo‐excitation and emission in highly oriented LCCPs. Also highlighted in Figure S4b, Supporting Information is a nearly identical vibronic spectral lineshape between the 22.5° (and 0°)‐polarized PL spectra of Film II and the non‐polarized PL spectrum of Film I, implying a common origin of PL emission in the two films, that is, the radiative decay of emissive excited intrachain state in the fully‐aligned nematic monodomain Film II and the LC‐assembled polymer nanocrystals in the non‐aligned polydomain Film I. These findings also support non‐radiative FRET funneling from the amorphous host to polymer nanocrystals in the nematic polydomains; and therefore, suppressed emission from the major amorphous matrix.

By taking the *R*_SW/LW_ ratio at the 0–0 (536 nm) and 0–2 (570 nm) vibronic peak as an estimation of the relative weighting of intrachain emission in a non‐polarized PL spectrum, we can underline a proportional relationship between the PLQE of all comparable F8BT films and the corresponding *R*_SW/LW_ in in Figure 3f. For the spin‐coated non‐LC Film 0, highly twisted chain conformations and highly localized BT‐to‐BT interchain interactions explain the observed dominance of the 0–2 vibronic transition band (thus, the smallest *R*_SW/LW_) and the lowest PLQE. The highest PLQE and greatest fraction of intrachain emission obtained for the nonaligned nematic polydomain Film I can be ascribed to the establishment of a self‐doped host–guest system, which combines the benefits of optimal absorption of non‐polarized photon energy, highly efficient host‐to‐guest FRET, dominant intrachain emission, and deactivating traps and energy losses associated with a major fraction of disordered F8BT chains. Even though the monodomain induces an additional emissive interchain species with an *H*‐aggregation character, a spatial averaging of this weakly emissive interchain species and the efficient intrachain emission lead to only intermediate *R*_SW/LW_ and PLQE under non‐polarized excitation. When the intrachain emission is separated by detecting PL signal at *θ*_Em_ = 0°, the *R*_SW/LW_ ratio is found to be up to ≈2.11–2.15 in the best ordered fully‐aligned LC monodomain thin films of F8BT (Figure S6, Supporting Information), pointing to a near‐unity PLQE for its intrachain emission.

Taking all of the structural and photophysical spectral information into account, we rationalize i) that a combination of 3D host‐to‐guest FRET funneling and J‐aggregated intrachain emission in F8BT nanocrystals governs the desirable PL properties in the self‐doped F8BT nematic polydomain glass film, ii) that high‐quality chain orientation in the extended nematic monodomains brings about high anisotropy in charge‐carrier transport and polarized absorption/emission that are inaccessible in the non‐LC and non‐aligned nematic polydomain analogues, whilst also promoting a new emissive excited interchain species that leads to an orthogonal polarized 0–1 vibronic PL emission band and reduction in the spatially‐averaged PLQE because of the *H*‐aggregation character and inseparable feature from intrachain emission, and iii) that the structural and energetic disorder in the spin‐coated non‐LC Film 0 and the major amorphous matrix of the nonaligned nematic polydomain Film I play distinct roles in the overall PL performance: the amorphous F8BT chains still dominate the optical and photophysical properties in Film 0, but the LC alignment upgrades the PLQE and effective defect tolerance of the nematic polydomains. Moreover, disordered F8BT chains in the non‐LC Film 0 result in a wider 0–2 vibronic PL band (e.g., full width at half maximum [FWHM] = 71 nm) than for the remaining non‐aligned nematic‐phase F8BT chains (FWHM = 46 nm) in the fully‐aligned monodomain Film II (c.f. Figure S7, Supporting Information), suggesting that the LC‐alignment mitigates energetic disorder in the donor matrix. Based on the *R*_SW/LW_ data obtained by fitting the 0–2 vibronic PL transition band in the non‐aligned nematic F8BT chains (Figure S7a, Supporting Information), we extrapolate the PLQE of “pure amorphous” nematic F8BT to be ≈15% (the red star in Figure 3f). This presents a lower PLQE value by a factor of ≈5 and ≈7 compared respectively with F8BT intrachain emission in LC multidomains and monodomains; hence, agreeing with the *H*‐aggregation character in the amorphous nematic F8BT.

### 2.4 Polarized Micro‐PL Spatial Spectral Mapping

**Figure 4:**
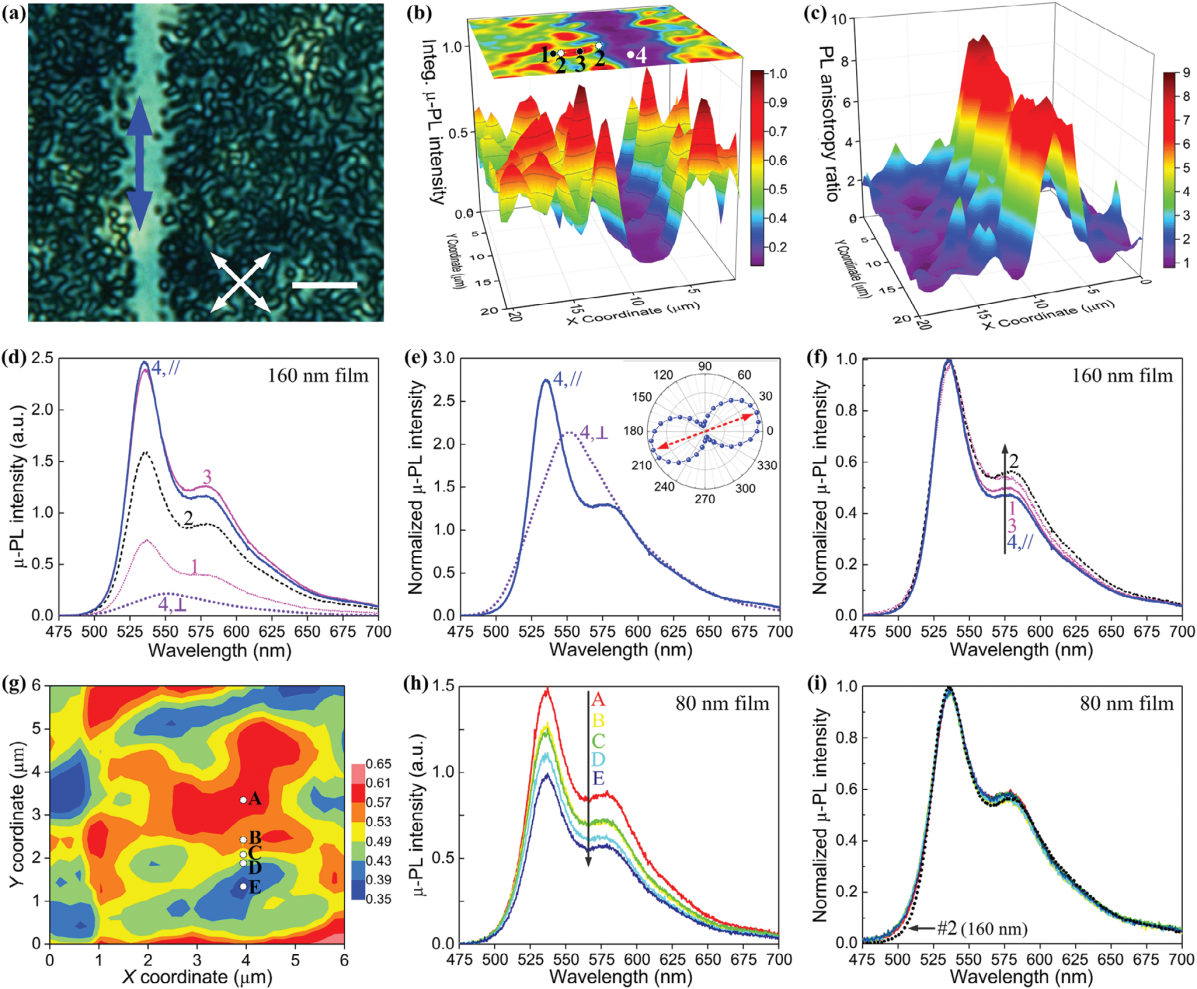
Polarized µ‐PL spectral characterization of a–f) a spatially patterned 160 nm‐thick and g–i) a nonaligned 80 nm‐thick F8BT nematic glass film. For the sample region plotted in (a–f), UV‐alignment of an SD1 layer has been patterned with a photomask consisting of an aligned channel against a non‐aligned background; the resulting F8BT alignment direction in the channel region is indicated by the double‐headed blue arrow in the polarizing optical micrograph (a) recorded from the same spatially‐patterned F8BT film (horizontal scale bar: 6 µm). b) 3D color‐coded µ‐PL map and a contour plot of the normalized total µ‐PL intensity as a function of the in‐plane location and c) corresponding 3D map of the spatial variation of the integrated PL anisotropy ratio. d) Polarized µ‐PL spectra and e,f) corresponding peak‐normalized µ‐PL spectra acquired at the different locations in the polydomain region (labelled as #1 – #3 in [b]) and also at the aligned line (#4). The µ‐PL spectra in (e) were normalized by the 0–3 vibronic peak intensity and those in (f) by the 0–0 vibronic peak. For scanning point #4, the perpendicular (⊥) and parallel (//) polarized µ‐PL spectra were recorded with emission collection polarization perpendicular and parallel, respectively, to F8BT transition dipole moment. The inset in (e) is a polar plot of the normalized total µ‐PL intensity from location point #4 versus the angle between the emission collection polarization and the chain alignment direction. g) A contour plot of the normalized total µ‐PL intensity across the polydomains in a representative nonaligned 80 nm‐thick F8BT nematic glass film. h) Polarized µ‐PL spectra and i) corresponding normalized PL spectra for points A, B, C, D, and E labeled in (g). The normalized µ‐PL spectrum from the domain boundary (the thick black dotted curve from location #2 in [b]) in the 160 nm‐thick nonaligned background was plotted in (i) for comparison.

High‐resolution spatial definition of the chain‐orientation, microstructural anisotropy, and concomitant energy landscape can be used to tailor the photophysical properties in the different types of F8BT films. The specific extent of structural inhomogeneity and energetic disorder, on the other hand, is extremely sensitive to the probing location and length scale of the relevant photophysical measurements. In order to elucidate the effect of high‐quality chain alignment and self‐assembled LC textures on the localized emission properties (e.g., polarized PL spectra and vibronic spectral lineshape, PL intensity anisotropy), systematic polarized micro‐PL (µ‐PL) measurements are carried out on an SD1‐aligned, spatially‐patterned 3 µm‐thick F8BT line against the nonaligned polydomain background in the same 160 nm‐thick F8BT nematic glass film sample. Spatial patterning of polymer chain orientation in the sample, as shown in the bright‐state POM in **Figure** **4**a and a continuous transition between the bright‐ and dark‐state in Movie S1, Supporting Information when rotating the spatially‐patterned F8BT nematic film sample in the plane of the substrate, is created using UV‐alignment with a photomask to illuminate a continuous SD1 layer before accessing the high‐temperature nematic phase to selective alignment of the overlying F8BT film (see Experimental Section and Figure S8, Supporting Information for more details). It is demonstrated that 2–4 µm feature sizes are readily generated in the photo‐masked chain‐orientation patterns against a nonaligned background in the correspondingly photoaligned F8BT nematic glass film.

All polarized µ‐PL spectra are acquired using a 405 nm excitation source with fixed beam polarization parallel to the chain‐orientation direction (i.e., *θ*_Ext_ = 0°) in the UV‐aligned line (along the *y*‐axis in Figure 4b,c). The emission is separated using an analyzer with polarization parallel (PL*_//_*) and perpendicular (PL*_⊥_*) to the transition dipole moment direction of the highly oriented F8BT chains in the patterned line region. By rotating the emission collection polarization in the film plane during acquisition of the polarized µ‐PL spectra within the aligned line, the direction of F8BT transition dipole moment (marked by the red dashed arrows in the insert of Figure 4e), where µ‐PL intensity from the aligned F8BT line is maximized under the fixed excitation, is found to align at *θ*_Em_ ≈20° relative to the chain‐orientation direction in the aligned channel. This deviation angle is close to the experimental and computational results of 20–22°.^[^ ^38^, ^39^ ^]^ A 3D rendered map (bottom of Figure 4b) and a 2D color‐coded image (top of Figure 4b) are included to help visualize the spatial variation of the integrated PL*_⊥_* spectral intensity in the aligned line and the nonaligned polydomain background (emission from the background was collected with the same excitation and analyzer polarization used to record the PL*_⊥_* spectra of the aligned line); the corresponding 3D mapping of the integrated *PL_//_* intensity from the same region of the sample is provided in Figure S9a, Supporting Information. The nonaligned polydomain background displays an irregular variation in the domain size/shape and µ‐PL intensity along both the *x*‐axis and *y*‐axis, revealing a random distribution of the chain orientation across different nematic polydomains.

Figure 4c further illustrates relatively small values of integrated PL anisotropy, that is, = ∫PL*_//_* / ∫PL*_⊥_*, from the nonaligned nematic polydomain background compared to the much larger and more uniform anisotropies in the photoaligned F8BT channel. The highly oriented F8BT chains in the aligned line collectively induce large integrated anisotropies of ≈9 due to the combined benefits of LC alignment and high‐quality photoalignment, especially those that involve a slight enhancement of the (intrachain) PL*_//_* intensity but a noticeable reduction in the interchain‐emission‐dominated PL*_⊥_* intensity.^[^ ^40^ ^]^ Inspection of the spatial variation of PL anisotropy across the LC‐assembled polydomain background (see also Figure S9b, Supporting Information) estimates the average domain size to be 3.5–5 µm by reflecting a 90° difference of chain orientation formed in nematic polydomain domains, which therefore tallies with the irregular polarized confocal fluorescence images shown in Figure 1b. For comparison, the averaged domain size of ≈2 µm across the POM images of the nonaligned polydomain Film I relies on a 45° difference in chain orientation.

To visualize the spatially resolved vibronic PL spectral structures in the in‐plane patterned sample of nematic F8BT, Figure 4d–f (and Figure S9c,d, Supporting Information) show the polarized µ‐PL spectra recorded from different locations across the representative domains in the nonaligned polydomain background (location #1 – #3) and in the photoaligned monodomain channel (location #4), as labeled in Figure 4b. All of these highly localized µ‐PL spectra should be dictated by the highly oriented F8BT chains or nanocrystals, given that emission of the disordered F8BT chains in the nonaligned polydomain film is effectively suppressed by nonradiative donor‐to‐acceptor FRET funneling. Altogether, our polarized µ‐PL spectral results unravel similar spectral lineshapes in the PL*_//_* and PL*_⊥_* spectra collected from the three probe locations in the nonaligned polydomain background, except for the domain boundary which (location #2) exhibits the best resolved (0–2) vibronic PL peak at ≈580 nm and the biggest PL peak intensity ratio between the 0–2 and 0–0 vibronic transition, *P*_(0−2)/(0−0)_. The *P*_(0−2)/(0−0)_ and µ‐PL intensity are generally correlated such that the higher the µ‐PL intensity is from a recorded location, the lower the *P*_(0−2)/(0−0)_ ratio in the µ‐PL spectra is for the same scanning location. Among all polarized µ‐PL spectra, the PL*_//_* spectrum from location #4 in the photoaligned channel manifests the highest PL intensity at the 0–0 vibronic peak but the lowest *P*_(0−2)/(0−0)_. In addition, these µ‐PL spectral findings agree with the shown relationship between *P*_(0−2)/(0−0)_ and PL intensity from the macroscopic PL spectra in Figure 3c,e.

In contrast to the analogous µ‐PL spectral lineshapes recorded from the three typical locations in the nonaligned polydomain background, a striking PL‐spectral separation of two orthogonally polarized PL components originating from emissive excited intrachain and interchain species is presented in Figure 4e for the photoaligned nematic monodomain line. Although the PL*_//_* spectra from location #4 can resemble the vibronically‐structured µ‐PL spectra of the nonaligned polydomain background (but with a slightly lower *P*_(0−2)/(0−0)_ ratio that is equivalent to a greater Huang–Rhys parameter and more rigid molecular geometry of the aligned chains), the relatively featureless PL*_⊥_* spectrum from location #4 peaks at ≈553 nm and has a much lower PL intensity than the PL*_//_* spectrum, having a PL intensity ratio of 15.4 at the 0–0 vibronic peak. When normalized by the 0–3 vibronic PL peak intensity, the PL*_//_* and PL*_⊥_* spectra from location #4 in Figure 4e display distinct 0–0 and 0–1 vibronic PL peak intensities; the peak‐normalized 0–1 vibronic PL peak in the PL*_⊥_* spectrum shows a larger intensity than that on the corresponding PL*_//_* spectrum, indicating that the PL*_//_* and PL*_⊥_* emission from the aligned F8BT at location #4 originate from two different emissive excited electronic states whose radiative decay can be polarized along two directions. The PL*_//_* spectrum is dictated by intrachain PL emission that is shown to linearly polarize along the direction of the F8BT transition dipole moment, whereas the PL*_⊥_* spectrum is formed mainly due to contributions of the emissive excited interchain species with its PL polarization direction being perpendicular to the chain alignment direction (i.e., along the interchain direction).^[^ ^41^ ^]^ The former indication is helpful to explain the presence of a projected 0–0 vibronic PL transition band in the perpendicular collected (*θ*_Em_ = 90°) PL spectrum of the fully‐aligned F8BT monodomain Film II (Figure 3e) because in this case, the macroscopic emission is selected with an orthogonal polarization angle relative to the chain alignment direction, which is still at a deviation angle of ≈20° from the direction of the transition dipole moment of the photoaligned F8BT chains in the extended nematic monodomain. The polarization‐dependent separation phenomenon between the two orthogonal polarized PL components is regarded to be a result of the anisotropic structural ordering preserved in the SD1‐aligned F8BT nematic monodomain. In particular, the additional interchain emission identified in the highly oriented F8BT monodomain is expected to be structurally allowed by the remarkably elongated interchain conjugation length and the associated formation of long‐range BT‐to‐BT electronic delocalization and strong interchain coupling. However, the minor polymeric nanocrystals dispersed in the amorphous chromophoric host in the nonaligned nematic polydomain Film I would trigger isotropic FRET, 3D excitation energy concentration, and no additional polarized emissive interchain species.

Another important finding from Figure 4d–f is that the domain boundary regions exhibit the best resolved 0–2 vibronic transition peaking and the greatest *P*_(0−2)/(0−0)_ ratio at ≈580 nm, both of which can be potentially ascribed to enhancement in interchain FRET funneling and structural order of F8BT nanocrystals. The domain boundaries that serve to interface adjacent nematic domains accommodate a higher extent of structural disorder than the domain interiors in order to smooth the heterogeneity between adjacent domains, and thus, are believed to incorporate a larger fraction of amorphous‐phase F8BT (or a decreased inclusion of polymer nanocrystals). This low accommodation of the acceptor elements can be attributed to a more favorable situation in which each F8BT nanocrystal acceptor couples with relatively more peripheral donor elements. By assuming that the donor‐to‐acceptor FRET process in the self‐doped host–guest system of mesophase F8BT is more efficient to induce the lower‐energy 0–2 vibronic PL transition band than the 0–0 transition, a tendency of incorporating a higher level of disordered F8BT would result in a larger *P*_(0−2)/(0−0)_ value. A similar change trend is observed in Figure 4g–i among the nematic polydomains in a nonaligned 80 nm‐thick nematic polydomain Film I, demonstrating a larger yet uniform *P*_(0−2)/(0−0)_ and a slightly blue‐shifted 0–2 vibronic µ‐PL peak relative to that of the nonaligned 160 nm‐thick polydomain background. In other words, additional PL contribution from an increased *P*_(0−2)/(0−0)_ ratio may correlate with the occurrence of more efficient interchain exciton migration owing to lower energy losses over a shorter interchain distance before the radiative decay. Accordingly, improved PLQE up to ≈70–72% is determined in various nonaligned but thinner (80 nm‐thick) F8BT nematic polydomain Film I; a reduction in film thickness results in a smaller spatially‐averaged domain size across the nematic polydomains, and thus, a slightly larger fraction of domain boundary regions and red‐shifted absorption spectra would correspond to larger spectral overlapping for more efficient FRET (Figures S10 and S11, Supporting Information). Based on the minimization of the energy of domain bulk against domain boundary energy, we arrive at a power law scaling of the spatially‐averaged nematic polyamine domain size (*w*) (*w* ∝ *D^n^*) with the film thickness (*D*), which has a nearly linear exponent of *n* ≈1.14 (see details in Section SIII, Supporting Information).

The thickness dependence of the F8BT dichroic ratio and the LC textures in the fully‐aligned F8BT nematic glass films are shown in Figure S12, Supporting Information. It can be seen that F8BT chain orientation arising from the UV‐aligned SD1 commanding layers is favored for the glass films with thicknesses in the range ≈100–300 nm, in terms of >10 dichroic ratios. The maximum dichroic ratio is 12.3 at a film thickness of ≈190 nm, which reaches the theoretical upper‐limit of dichroic ratio (*S* = 1) by considering a deviation angle of 20–22° between F8BT chain axis and the direction of transition dipole moment. Due to the reduced aligning force imposed by the photoalignment layer, a further increase in F8BT film thickness can constantly lower the overall F8BT alignment quality averaged across the whole thickness of the aligned nematic glass films; we nevertheless demonstrate that the high‐quality chain‐orientation occurs for film thicknesses similar to those commonly used in a wide range of device structures.

### 2.5 Polarization‐Dependent Spectral Separation and Time‐Resolved PL Decay

**Figure 5:**
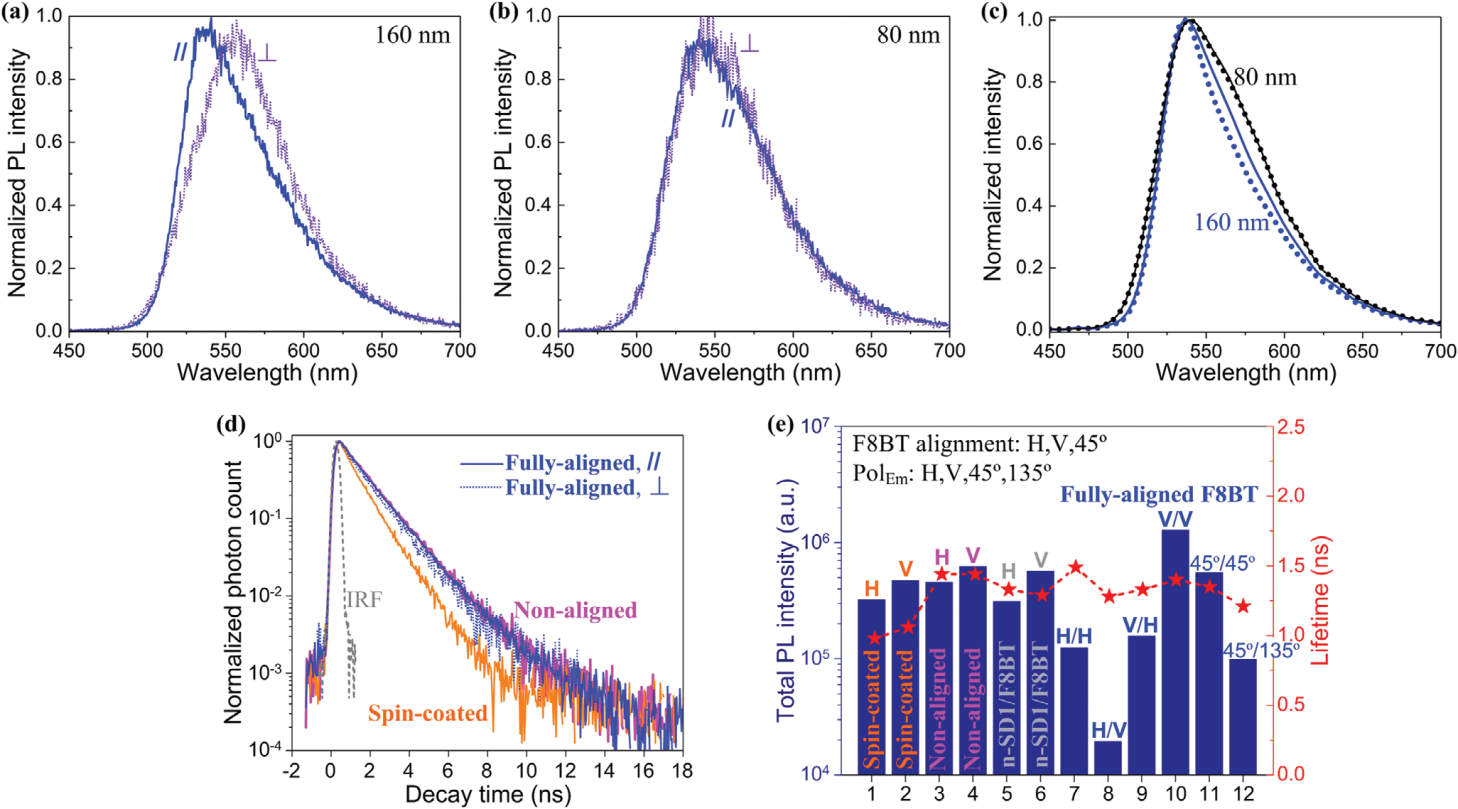
a,b) Area normalized time‐integrated PL spectra recorded from the fully‐aligned F8BT nematic glass films with a thickness of 160 nm (a) and 80 nm (b). The excitation laser remained linearly polarized parallel to the chain alignment direction in the photoaligned F8BT films; emission was collected with polarization parallel (*//*) or perpendicular (*⊥*) to the excitation polarization. c) Peak‐normalized shortpass filtered PL*_//_* (solid curve) and PL*_//_* − PL*_⊥_* (dotted curve) spectra for a photoaligned 80 nm (black curve) and 160 nm (blue curve) F8BT film. Here, the PL*_//_* − PL*_⊥_* spectra were compared to elucidate the occurrence of spectral separation in the two photoaligned nematic films by excluding the polarization‐independent PL contribution arising from any randomly oriented F8BT chains in the aligned film. d) Peak‐normalized PL transients recorded from a spin‐coated non‐LC (brown), a nonaligned LC (pink), and a fully‐aligned F8BT nematic film (blue) recorded from the parallel (solid blue) and perpendicular (dotted blue) polarization configuration. All F8BT films plotted in (d) were 80 nm in thickness; the grey dotted curve shows the instrument response function (IRF). e) The total PL intensity (bars) and single‐exponential fitting lifetime (stars) for four comparative F8BT films of thickness 80 nm: spin‐coated non‐LC (brown label), nonaligned LC (pink), a bilayer consisting of a non‐aligned continuous SD1 (n‐SD1) commanding layer and a non‐aligned overlaying F8BT nematic glass (grey), and fully‐aligned nematic glass (blue). The excitation laser used to acquire the as‐shown polarized PL transients remained linearly polarized along the vertical direction in the vertical plane. The labels in (e) were defined as the following: the first angle or letter (*V* for the vertical or *θ*_Ext_ = 0° direction, *H* for horizontal or *θ*_Ext_ = 90°) represents the chain alignment direction locked in the photoaligned F8BT film, and the second label (*V, H*, 45°, 135°) denotes the direction of emission collection polarization (Pol_Em_).

The type of nematic LC textures (polydomain or monodomain) preserved in the SD1‐aligned F8BT films is further unveiled to play a deciding role in F8BT emission lifetime and also in whether or not the PL*_//_* and PL*_⊥_* spectra from an F8BT film can be separated. **Figure** **5**a,b shows the time‐integrated PL*_//_* and PL*_⊥_* spectra from an SD1‐aligned F8BT film (by a UV‐aligned continuous SD1 layer) with 160 and 80 nm thickness, respectively. The fully‐aligned 160 nm F8BT nematic film exhibiting a monodomain LC texture also shows a clear polarization‐dependent PL spectral separation. In contrast, the aligned 80 nm nematic film displays a polydomain texture and no clear change in the lineshape for both the PL*_//_* and PL*_⊥_* spectra (see Figure 5c for PL*_//_* − PL*_⊥_* spectra). These opposite spectral separation cases elucidate a strong structure–property relationship in the SD1‐aligned F8BT nematic films: the locked‐in LC texture, and more importantly, the absence and presence of domain boundaries in the F8BT nematic films can dictate the occurrence of polarization‐dependent PL spectral separation.

The extracted PL lifetime (*τ*) and rate constants for the radiative decay (*k*_R_) and nonradiative decay (*k*_NR_) for the spin‐coated non‐LC F8BT (Film 0), the nonaligned nematic (Film I), and the fully‐aligned (Film II) nematic films of F8BT are summarized in **Table** **2**; Figures S13 and S14, Supporting Information. The rate constants are calculated based on the equations: 1/τ = *k*_R_ + *k*_NR_ and PLQE = *k*_R_/(*k*_R_ + *k*_NR_). The spin‐coated non‐LC reference film exhibits the shortest lifetime (1.02–1.16 ns) and the lowest PLQE, which can be ascribed to the prevalence of the nonradiative decay and traps associated with the adoption of highly twisted chain conformations and a disordered microstructural arrangement. Locking in the nematic phase increases the (intrachain) PL*_//_* lifetime by 30–40%, but the uniaxial polymer photoalignment by UV‐aligned SD1 layer does not remarkably enhance the lifetime further because all evaluated PL*_//_* spectra herein are dominated by the stronger intrachain PL emission with linear polarization along an in‐plane direction that is not parallel to the photoaligned chain orientation. We find that the PL*_//_* spectra have a longer lifetime than PL*_⊥_* in the two fully‐aligned F8BT nematic glass films; the PL*_⊥_* emission and its shortened lifetime could be originated from lossy interchain exciton transport and slower relaxation dynamics of the emissive interchain species. The similar fluorescence lifetimes of these measured PL transients over the emission band at different detection wavelengths (c.f. Figure S15, Supporting Information) reveal that the overall lifetime of both PL*_//_* and PL*_⊥_* emission does not clearly alter with the detection wavelength and photon energy, implying that the PL*_//_* and PL*_⊥_* spectra are likely governed by only one emissive species, albeit their different vibronic PL spectral structures. Taking all lifetime results together, we would rationalize that the formation and preservation of a polydomain LC texture in F8BT glass films, irrespective of the quality of polymer photoalignment, effectively suppresses energy losses induced by the nonradiative decay and quenching of traps existing in a major fraction of the amorphous matrix of F8BT chains, for example, in terms of suppressing the nonradiative decays via short range host‐to‐guest FRET funneling or making use of the remarkably efficient radiative decay of more extended F8BT chains in the self‐assembled polymer nanocrystals. This suppression effect becomes mitigated in the fully‐aligned monodomain nematic film due to a spatially averaging effect of a polydispersity‐defined variation of polymer chain lengths, whereby the less efficient transport and slower decay rate of the additional interchain excited states come into play and compete with the intrachain excitonic dynamics.

**Table 2:** Summary of extracted lifetime (*τ*) and rate constants for the radiative decay (*k*_R_) and nonradiative decay (*k*_NR_) in the spin‐coated non‐LC F8BT film (Film 0), as well as in the nonaligned (Film I) and fully‐aligned (Film II) F8BT nematic films with different thicknesses.

	80 nm‐thick F8BT films			160 nm‐thick F8BT films		
	Film 0	Film I	Film II	Film 0	Film I	Film II
LC texture	Non‐LC	Polydomain	Polydomain	Non‐LC	Polydomain	Monodomain
*τ* [ns]	1.02 ± 0.04	1.44 ± 0.04	1.38 ± 0.07	1.16 ± 0.04	1.47 ± 0.04	1.40 ± 0.04
*k*_R_ [10^8^ s^−1^]	2.9 ± 0.1	4.7 ± 0.1	4.4 ± 0.2	3.0 ± 0.1	4.6 ± 0.1	3.6 ± 0.1
*k*_NR_ [10^8^ s^−1^]	6.9 ± 0.3	2.2 ± 0.2	2.9 ± 0.3	5.6 ± 0.3	2.1 ± 0.2	3.6 ± 0.3

## 3 Concluding Remarks and Outlook

This work systematically studies the structure–property relationship in solution processed light‐emitting LCCP films via fine‐tuning the structural ordering (order parameter ranging from 0 to ≈100%) by making use of the LC‐phase ordering and high‐quality photoalignment of chain orientation. Bringing structural order into the otherwise disordered F8BT films enables us to optimize the spatial distribution and interactions of physical structures and enable polarized photophysical properties. The PL polarization anisotropies in the photoaligned F8BT nematic monodomains are generally larger than the corresponding optical dichroic ratios because only partial chain segments contribute to emission but the whole backbones are active for optical absorption. Even for a nearly 100% order parameter, as demonstrated in the photoaligned F8BT nematic monodomains,^[^ ^40^ ^]^ the GIWAXS data indicate imperfect (herein estimated to be 60–73%) overall crystallinity, confirming that the degree of polymer crystallinity is also sensitive to the local arrangement of side‐chains and π–π stacking between backbones.

We demonstrate that the commonly‐used pristine (e.g., spin‐coated and nearly amorphous) conjugated copolymer layers tend to adopt exceedingly twisted chain conformations and possess a significant population of localized interchain interactions in order to minimize steric hindrance. This disordered structural configuration is fundamentally responsible for the absence of long‐range structural order, a dominance of the low‐energy vibronic transition bands in the PL spectra, the lowest PLQE, and shortest lifetime among all the comparative F8BT films measured in this study; a high degree of structural disorder also gives rise to a short‐ranged but strong electronic coupling between the excited state and the ground state; and thus, a shortened PL lifetime as well. The mesophase long‐range orientational ordering alone is adequate to facilitate the self‐assembly of a minor fraction of polymer nanocrystals (with the most extended chain conformations) in a pool of disordered LCCP chains. This self‐doping within the Schlieren polydomains of nematic F8BT then reconciles a bioinspired host–guest system to make use of the nonradiative 3D donor‐to‐acceptor FRET funneling, and eventually, efficient intrachain PL emission of F8BT nanocrystals. For this self‐doped host–guest system, there exists a sufficient spatial difference in the energy level of polymer (semi‐)crystals and that of the amorphous host, which facilitates favorable excitonic energy concentrations; and therefore, considerably enhances the overall PLQE and defect tolerance. The LC‐phase structural reorganization in the nematic F8BT polydomain film gives rise to PLQE values >70%, which approaches that of the theoretical upper limit of isolated chains in the solution of F8BT. The presented light‐harvesting and PLQE reinforcement mechanism (via engineering the physical structures of an LCCP) are somewhat analogous to the adaption of photosynthetic organisms to fluctuating sunlight via regulating the involvement of active smaller‐ and larger‐sized LHCs.

Macroscopic LC domain texture and relative weighting of LCCP nanocrystals in a nematic polydomain glass film play a key role in limiting the excitation energy transfer and emission efficiencies. Our polarized PL spectral mapping highlights a similar lineshape that exists between the nematic polydomains and the PL*_//_* spectra of the photoaligned monodomains. The domain boundary regions, where a greater fraction of the amorphous‐phase donor is accommodated to increase the donor/acceptor spectral overlapping, can be more favorable than the domain interior when it comes to enhancing FRET/PL efficiencies and suppressing the nonradiative decays associated with traps. Although the localized polymer nanocrystals take up a low weight fraction (Est. 5%) in the nonaligned nematic polydomain film, they govern the emission properties of the F8BT glass films by making the major amorphous matrix non‐emissive. Additional evidence for the creation of a self‐doped host–guest system is provided by the largest PLQE within the nonaligned F8BT nematic polydomain Film I and such a high PLQE is further augmented by reducing the spatially averaged domain size of self‐doped nematic polydomains. High‐quality chain orientation in the photoaligned F8BT monodomain films increases optical absorption strength along the chain alignment direction and generates exceptional anisotropies in the structural order and PL polarization anisotropy. The anisotropic in‐plane packing of the extended F8BT monodomain allows for an additional luminescent interchain species that exhibits a PLQE value seven times lower than that of the intrachain emission; these structural and coupling aspects are the basis for a linear decrease of PLQE with increasing chain‐alignment quality. Strong or long‐range electronic coupling, especially when involving *H*‐aggregation and close acceptor‐to‐acceptor contacts between adjacent copolymer chains, is accompanied by a considerably low PLQE, because in this case, the interchain energy transfer is vulnerable to traps with a tendency to arrive at red‐shifted charge transfer states.^[^ ^42^ ^]^ A substantial enhancement in polymer crystallinity of the highly‐oriented, large‐area‐extended F8BT nematic monodomains gives rise to the polarization‐dependent spectral separation of the two orthogonal emission components of distinct characters that are not possible in the traditional amorphous deposits, along with the broadened PLE spectra pointing to access to extra band‐edge and sub‐band electronic states. An interplay of mesophase self‐assembly and SD1 molecular templating effect in a LCCP glass film makes it possible to access the otherwise deeply trapped electronic states and the strong light–matter interaction regime.^[^ ^43^ ^]^

Topics for further study include: i) Use of ultrafast pump‐probe techniques such as phase‐cycled transient absorption (TA)^[^ ^44^ ^]^ and multidimensional (e.g., 2D electronic‐vibrational^[^ ^45^, ^46^, ^47^ ^]^) spectroscopic methods to separate the competing intrachain and interchain exciton relaxation pathways in self‐doped and photoaligned organic semiconductors to elucidate multi‐exciton interaction dynamics and polarization‐related coherence of involved electronic and vibrational coupling in the excited state. ii) Application of spatial patterning of high‐quality molecular photoalignment into other mesophase and LC/molecular hybrid systems to unlock the full potential of large‐area extended polymer and molecular (semi‐)crystals, such as via inscribing bespoke in‐plane patterns for refractive index modification and light confinement/processing, which may transform the design and manufacture of electrically pumped lasing diodes, planar optical cavities, and optical circuits and meta‐structures, alongside wiring with natural photosystems toward semi‐artificial photosynthesis and quantum‐coherent energy and information transfer.^[^ ^47^, ^48^ ^]^ iii) Combining the photoalignment and polymer self‐doping approach explored in the present study with external doping methods^[^ ^49^, ^50^ ^]^ could bring structural ordering into some high‐performing photoelectrocatalytic, thermoelectric, bioelectronic, and bio‐mimicking organic semiconductor systems.^[^ ^51^, ^52^, ^53^ ^]^

## 4 Experimental Section

### Materials and Film Preparation

The photo‐alignment layer material, SD1, was provided by DIC Corporation Japan and used as received. The F8BT polymer with molecular weight, *M*_w_ = 55 000 and polydispersity index, PDI = 2.3 was purchased from ONE‐Material Inc. The photoalignment layers were spin‐coated from SD1 solutions in anhydrous 2‐methoxyethanol (≥99.8%) onto pre‐cleaned Spectrosil substrate at 500 rpm for 5 s, then at 2000 rpm for 20 s using SD1 solution concentrations of 0.5 and 0.1 mg mL^−1^ for the fabrication of continuous and discontinuous SD1 layers, respectively. These discontinuous SD1 films were used to induce partial domain alignment in the overlying F8BT films in the nematic phase, while the continuous SD1 layers (<4 nm in thickness) were employed to fully align the overlying F8BT nematic films. After spin‐coating, the SD1 photoalignment layers were annealed at 110 °C for 10 min in ambient conditions to ensure solvent removal, which was followed by molecular alignment performed via uniform polarized UV light exposure as described below. F8BT was then spin‐coated onto the UV‐aligned continuous or discontinuous SD1 layers using the same filtered 30 mg mL^−1^ solution in anhydrous toluene. The thickness of the F8BT films was tuned by systematically varying the spin‐coating speed and duration. The F8BT films were vacuum‐dried for >1 h to remove excess solvent, and then subjected to thermotropic alignment as described below.

### UV Alignment of SD1 Photoalignment Layer

SD1 photoalignment layers were aligned by irradiating the samples in air with 5 mW cm^−2^ linearly polarized light (*λ* = 365 nm that is close to the absorption peak of SD1) from a Thorlabs CS2010 UV‐curing LED system equipped with a WP25M‐UB broadband wire‐grid polarizer. For spatial patterning of the alignment region in the SD1 alignment layer, polarized UV exposure was used in combination with a specially‐made photomask placed above the SD1 layer (with a spatially patterned silver surface facing the top surface of SD1 layer). The spatially defined photomask pattern was fabricated based on a standard photolithography technique for spatially patterning a silver‐coated (150 nm thickness) glass substrate (see also the fabricated silver pattern on the photomask in Figure S7, Supporting Information). The duration of the polarized UV irradiation for aligning the continuous and discontinuous SD1 layers was varied over a range from 3 to 20 min so as to produce varying levels of structural order in the SD1 layers, which were then utilized to produce the dichroic ratios in the overlying F8BT films, as shown in Figure 3b in the main text.

### Thermotropic Alignment of F8BT Films by UV‐Aligned/Patterned SD1 Commanding Layer

Thermotropic alignment of F8BT film samples that have been spin‐coated on top of an aligned/patterned, continuous/discontinuous SD1 photoalignment layer was carried out by fine‐controlling temperature using a Linkam THMS600 hotstage. A F8BT film sample was heated at 20 °C min^−1^ to 265 °C (into a disordered melt) before being slowly cooled from 3 °C min^−1^ to 250 °C (nematic phase), and then held for 10 min. Subsequently, the sample was rapidly quenched to room temperature by transferring the sample from the hot‐stage onto a copper bar. This quenching step could effectively prevent crystallization and thereby “freeze” the SD1‐aligned LC ordering of F8BT chains, yielding a highly oriented nematic glass film. Alternatively, quenching F8BT directly from its melt above the clearing temperature of ≈265 °C to room temperature resulted in nematic glassy‐phase F8BT films. All fabrication steps were carried out in a nitrogen atmosphere glovebox operating with <1 ppm moisture and oxygen content. The thicknesses of all F8BT samples were measured using a Dektak profilometer.

### Polarized µ‐PL Spectral Mapping

The µ‐PL spectra were recorded at room temperature with linearly polarized 405 nm, 150 fs, and 76 MHz excitation from a frequency doubled Ti:sapphire laser (refer to the schematic setup shown in Figure S1 and Section S1.5, Supporting Information). The spot size on the film samples was ≈1 µm diameter; the scanning step of the high precision *x*–*y* positioning stage was set as 200 nm in both *x* and *y* scanning directions.

### Time‐Resolved PL Decay

The excitation source was a 379 nm laser that was pulsed at 10 MHz and linearly polarized in the vertical direction. The monochromator grating and streak‐camera setup allowed for a time range of 20 ns (10K acquisition) and the collection of 288 nm emission wavelength range (432–720 nm) with 6 nm spectral resolution at each time point. The obtained PL transients were then integrated to record the kinetic traces of different PL regions. The F8BT film and collection polarizer for PL spectral selection were rotated separately as to tailor the relative direction between excitation polarization and emission collection polarization.

## Conflict of Interest

The authors declare no conflict of interest.

## Supporting information

Supporting Information

Supplemental Movie 1

Supplemental Movie

## Data Availability

The data that support the findings of this study are available in the Supporting Information of this article.
