# Supplementary material for: Fine‐Tuning the Microstructure and Photophysical Characteristics of Fluorescent Conjugated Copolymers Using Photoalignment and Liquid‐Crystal Ordering
Source: Adv Sci (Weinh). 2024 Aug 29;11(41):2407117. doi: 10.1002/advs.202407117 (PMC11538637; doi:10.1002/advs.202407117)
Supplement: Supplementary file 1 — Supporting Information [file ADVS-11-2407117-s003.pdf]

ADVANCED SCIENCE
Open Access

Supporting Information

for *Adv. Sci.*, DOI 10.1002/advs.202407117

Fine-Tuning the Microstructure and Photophysical Characteristics of Fluorescent Conjugated Copolymers Using Photoalignment and Liquid-Crystal Ordering

Yuping Shi, Katharina Landfester and Stephen M. Morris*

Supplementary Information

Fine-tuning the Microstructure and Photophysical Characteristics of Semiconducting Conjugated Copolymers Using Photoalignment and Liquid-crystalline Ordering

Yuping Shi, Katharina Landfester, and Stephen M. Morris*

Dr. Y. Shi, Prof. K. Landfester
Max Planck Institute for Polymer Research, Ackermannweg 10, Mainz 55128, Germany
*To whom correspondence may be addressed. E-mail: shiy@mpip-mainz.mpg.de

Dr. Y. Shi, Prof. S. M. Morris
Department of Engineering Science, University of Oxford, Parks Road, Oxford, OX1 3PJ, UK

Table of Contents

SECTION **I**. Supplementary Description of Experimental Method 2
SECTION **II**. Supplementary Figures and Discussions 4
SECTION **III**. Domain-size Engineering and Scaling vs F8BT Film Thickness 13
SECTION **IV**. Photoalignment of F8BT Nematic Films with Varying Thickness 15
SECTION **V**. PL Transients and Lifetime Results 17
SI References 20

SECTION I. Supplementary Description of Experimental Method

1.1 Grazing-incidence Wide-angle X-ray Scattering (GIWAXS) Characterization

GIWAXS measurements of the different types of F8BT films were carried out at the Surface and Interface Diffraction beamline (I07) at the Diamond Light Source (DLS) using a beam energy of 20 keV (0.62 Å) and a Pilatus2M area detector. The samples were probed while inside a vacuum chamber at a pressure of around 10^{-3} mbar with the MINERVA setup.[1] The sample-to-detector distance was 41.8 cm as determined via AgBeh calibration. Images were converted to 2D reciprocal space using the DAWN software package.[2]

1.2 Polarized UV-vis Absorption and Polarized PL Spectra

Polarised UV-vis absorption spectroscopy was carried out using a PerkinElmer Lambda 1050. A Glan-Thompson polarizer was mounted in front of the F8BT films to generate a linearly polarized incident beam. The relative orientation of the polymer chains to this incident light polarization was varied by rotating the F8BT film in the vertical plane on a rotation stage. Polarized PL spectra were carried out using Horiba FluoroMax-4 and acquired by mounting a Glan-Thompson polarizer in front of the F8BT films to align the excitation (λ = 450 nm) polarization and using a build-in rotatable polarizer in the vertical plane to control the direction of PL collection polarization.

1.3 PLQE Measurements: Non-polarized PLQE measurements were performed the Horiba Quanta-Phi diffusely-reflecting integrating sphere attachment of Horiba FluoroMax-4 using non-polarized 450 nm excitation. Three film samples were measured for each type of F8BT film and for each kind of film sample two measurements were carried out with the second test performed by rotating the film by 90° in the horizontal plane relative to the first measurement so as to study the effect of polymer chain alignment direction on PLQE. For the F8BT solution sample, four measurements were carried out with different cuvette positions in the integrated sphere. The PLQE values were then calculated using the methodology detailed in SI Ref. [3].

1.4 Polarised Micro-Photoluminescence Spectral Measurements

Micro-PL (μ-PL) spectral characterisation resolves both the PL intensity and spectrum at a given scanning pixel. The μ-PL spectral maps of LCCP films presented were recorded at room temperature with linearly polarised 405 nm, 100 fs, and 76 MHz excitation from a frequency-doubled Ti: sapphire laser, using the schematic setup illustrated in **Figure S1**. The excitation laser was focused on the top surface of an F8BT film by a 100× objective with a numerical aperture of *NA* = 0.7. The spot size of the incident laser beam focused on the sample was ≈1 μm in diameter. The polarisation of the incident light and the collected PL spectra were

controlled using a combination of linear polarisers and half-wave plates in front of the objective and the spectrometer, respectively. The excited PL emission was collected by the same objective, dispersed by a 0.3-m-long spectrometer with a 300 lines/mm grating and detected by a thermoelectrically cooled charge-coupled device (CCD). For alignment purposes, the sample was illuminated by a broadband visible light source (yellow path) and an image of the sample was then collected by a CCD camera. A 430 nm long pass filter was used to remove the excitation laser. For mapping of the polarised μ-PL spectra, the samples were held on a piezo-electric controlled platform and a scanning step size of 200 nm in both the *x* and *y* axes.

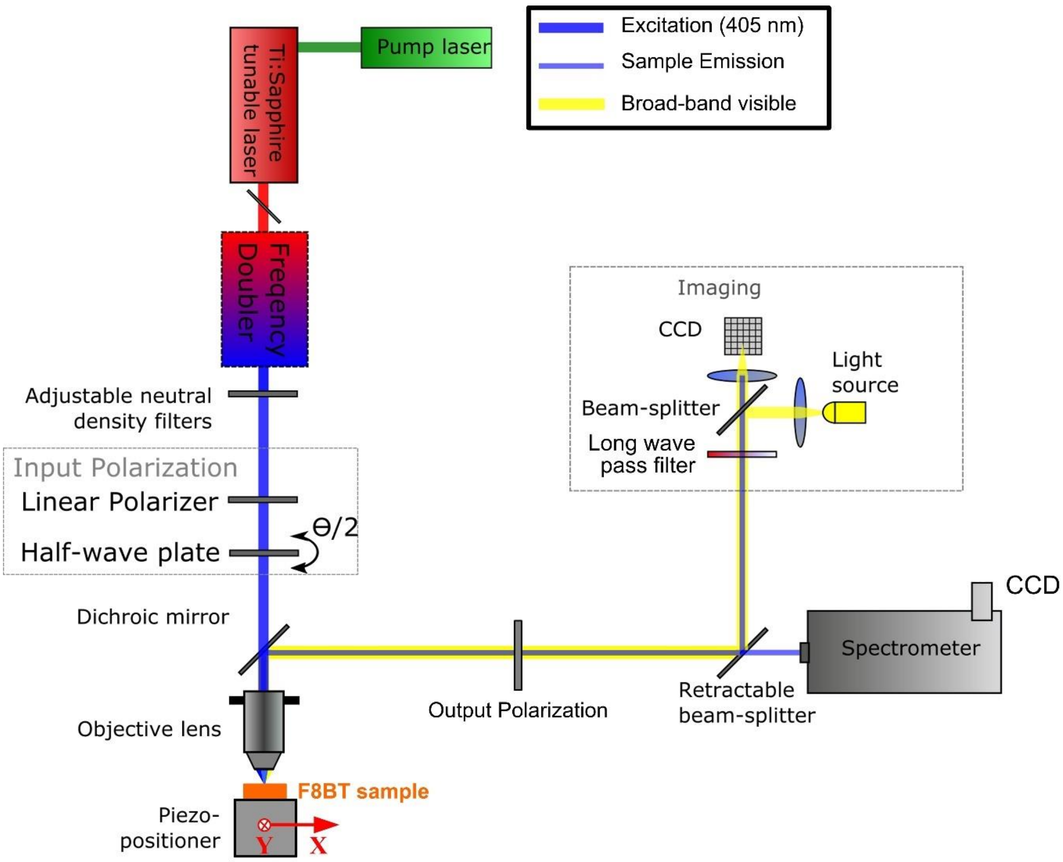

Figure S1. Experimental setup for the polarised μ-PL spectral mapping measurements.

SECTION II. Supplementary Figures and Discussions

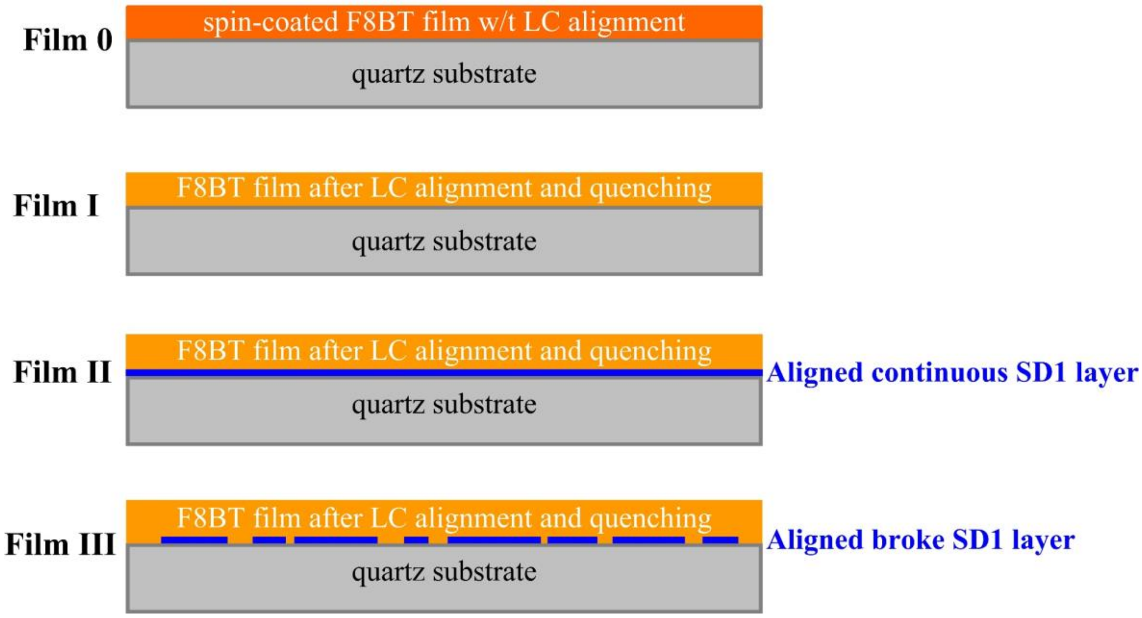

Figure S2. Illustration of the layer structure of the four kinds of F8BT films.

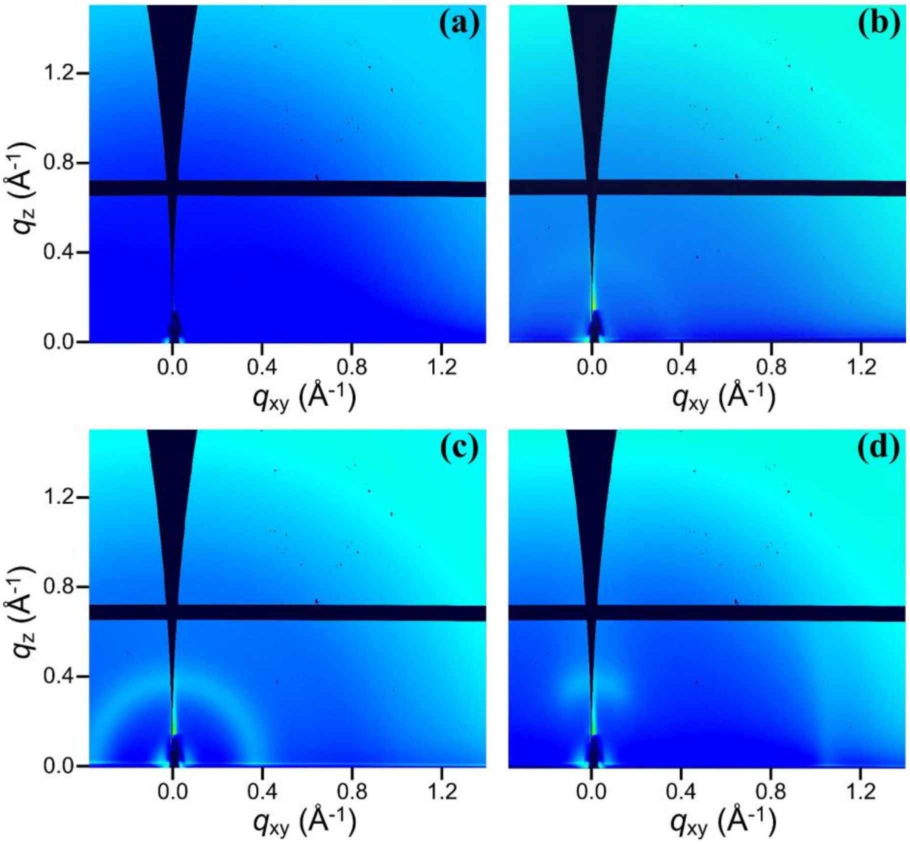

Figure S3. Raw GIWAXS data (i.e., before background and baseline subtraction) recorded from (a) a spin-coated non-LC F8BT film (*Film 0*), (b) a nonaligned F8BT nematic glass film (*Film I*), as well as (c - d) a fully-aligned F8BT nematic film (*Film II*) when the propagation of the incident X-ray beam is either aligned parallel (c) or perpendicular (d) to the chain alignment direction therein. All measured F8BT films are ~160 nm in thickness.

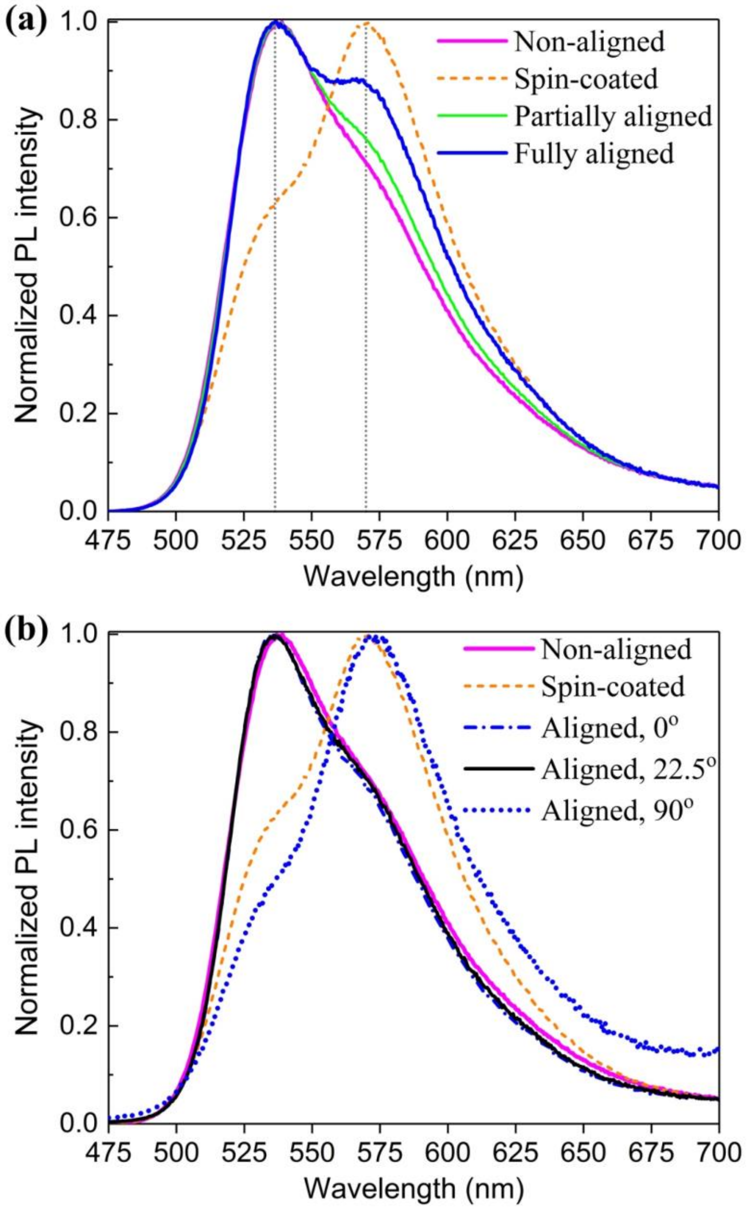

Figure S4. (a) Peak-normalized non-polarized PL spectra from the spin-coated non-LC F8BT film (*Film 0*), as well as the non-aligned (*Film I*), fully aligned (*Film II*) and partially aligned (*Film III*) F8BT nematic glass films. The vertical dotted lines indicate the location of the wavelength of the 0-0 and 0-1 vibronic PL peaks. All measured films herein are 160 nm thick, and PL measurements were carried out at room temperature. (b) Direct comparison of the peak-normalized non-polarized PL spectra of both the spin-coated non-LC film and the nonaligned nematic glass films with the polarised PL spectra collected from the fully-aligned F8BT nematic monodomain glass film using the three emission collection polarization angles: θ_{Em} = 0° ($PL_{//}$ spectrum), 22.5° (along the direction of optical transition dipole moment) and 90° ($PL_{\perp}$ spectrum), as labelled in Figure 3e in the main text.

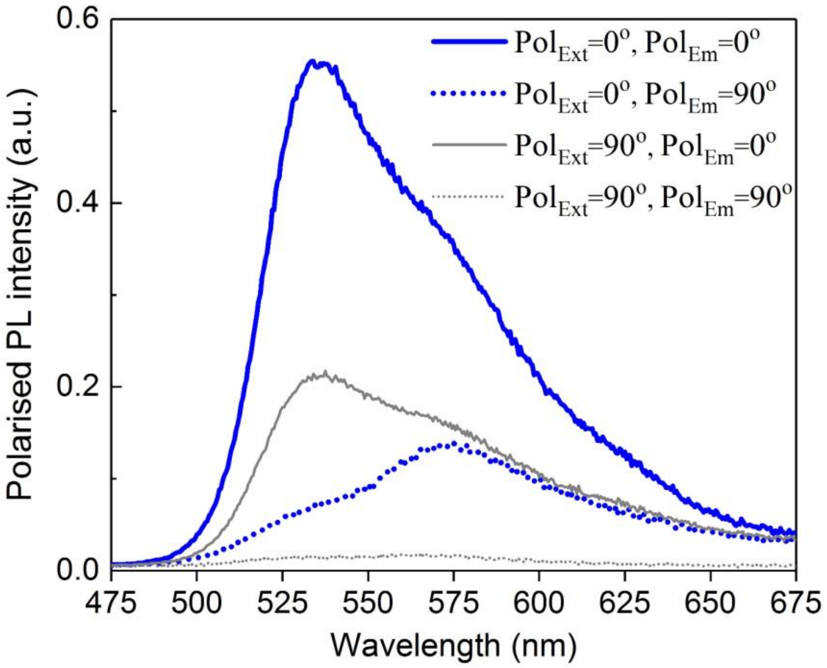

Figure S5. The polarised PL spectra collected from the fully-aligned F8BT nematic monodomain film (160 nm thickness) using the four combinations of the excitation polarization angle (Pol_{Ext}) and emission collection polarization angle (Pol_{Em}). Here, Pol_{Ext} and Pol_{Em} were defined relative to the chain alignment direction in the oriented film.

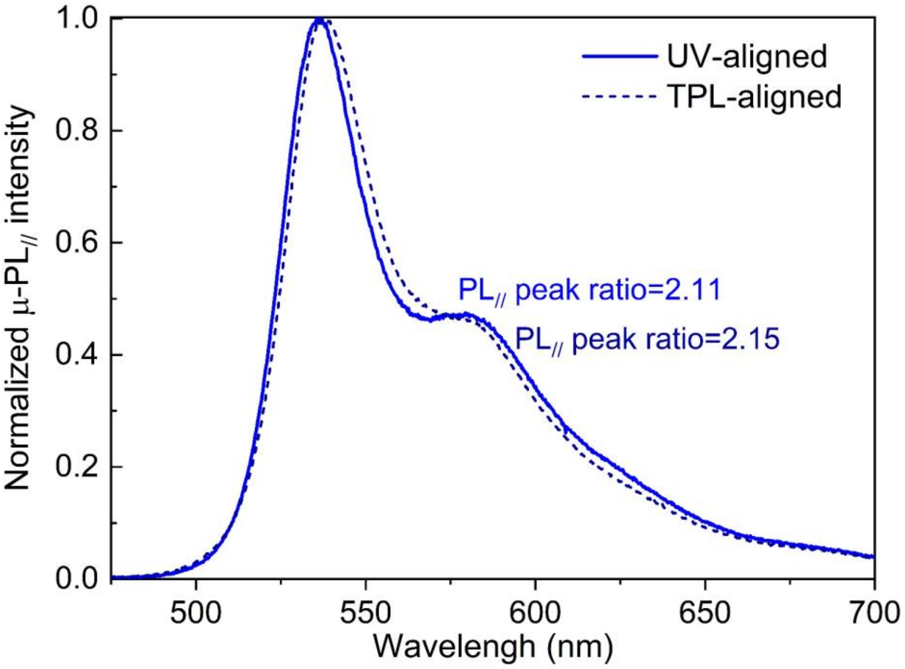

Figure S6. Micro $PL_{//}$ spectra in our best UV-aligned F8BT nematic monodomain film (film thickness = 190 nm) and also in two-photo laser (TPL) aligned nematic monodomain pattern (size: 100×100 µm square, see more information in Ref. [40] and [27] cited in the main text). Vibronic PL peak ratios of >2 were obtained from these two µ-$PL_{//}$ spectra.

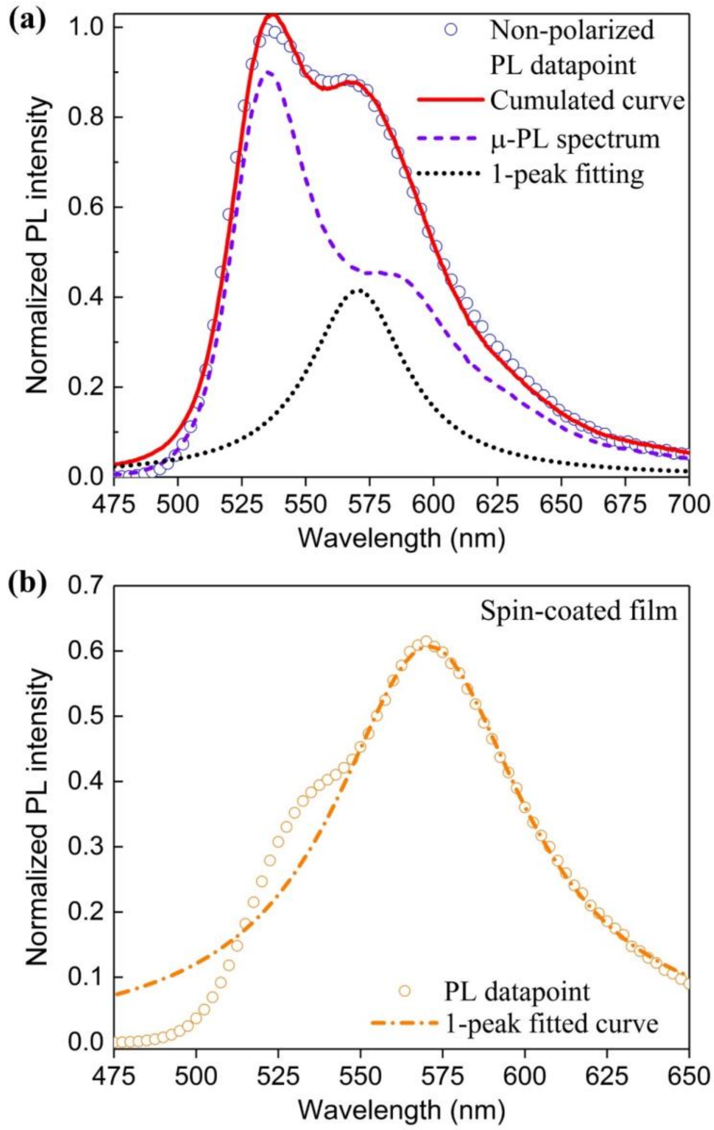

Figure S7. (a) Illustration of two-component fitting (the solid red curve) of the non-polarised PL spectrum (blue open circle) collected from the fully-aligned 160 nm F8BT nematic monodomain glass film shown in Figure 3c in the main text. One PL component (dotted black curve) used a Lorentzian peak centered at 570 nm with its width and peak intensity being set free during the implementation of the least-square fitting, the other PL component was determined by summating the two orthogonal polarised micro-PL spectra (shown in Figure 4d for location #4 in the photoaligned line), namely, the lineshape of the PL spectrum marked by the blue dashed curve herein is the normalised intensity of the $PL_{//} + PL_{\perp}$ from the photoaligned F8BT monodomain line (Location #4) of the same film thickness, but with 20% line boarding of both $PL_{//}$ and $PL_{\perp}$ spectra as to obtain the best fitting of the non-polarised macroscopic PL spectrum. (b) One (Lorentzian)-peak fitting of the longer-wavelength optical transition band on the non-polarised PL spectrum collected from the spin-coated non-LC reference film (*Film 0*).

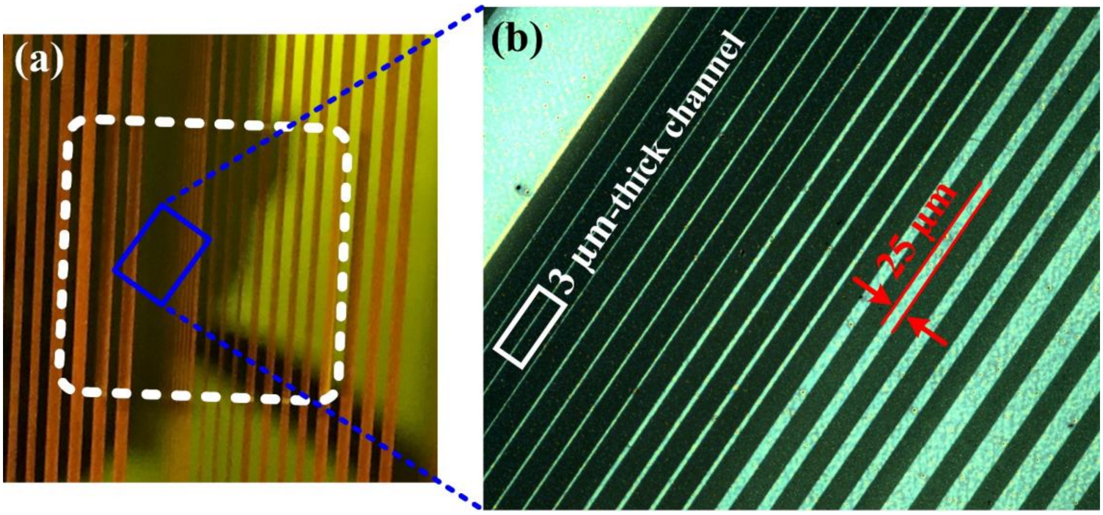

Figure S8. Illustration of the photo-masked UV-alignment method used to spatially define a bespoke alignment pattern in the SD1 alignment layer and an overlying F8BT glass film. (a) An optical photo demonstrating a large photomask placed on an SD1-coated quartz substrate (denoted by the dashed white square; substrate size: 25 mm × 25 mm) in the process of polarised UV illumination of the SD1 photoalignment layer. (b) Bright-state polarized optical micrograph of the ensuing chain-orientation pattern in a selected region, as labelled by the blue rectangle in (a), in the thermotropically aligned and then quenched overlying F8BT glass film. In (b) there are five groups of 4 × channels with a line width of 3.0 µm, 5 µm, 10 µm, 25 µm, and 50 µm; the solid white rectangle locates the UV-aligned 3 µm-thick F8BT channel against the nonaligned nematic glass background, both of which have been particularly investigated using the polarised µ-PL mapping measurements.

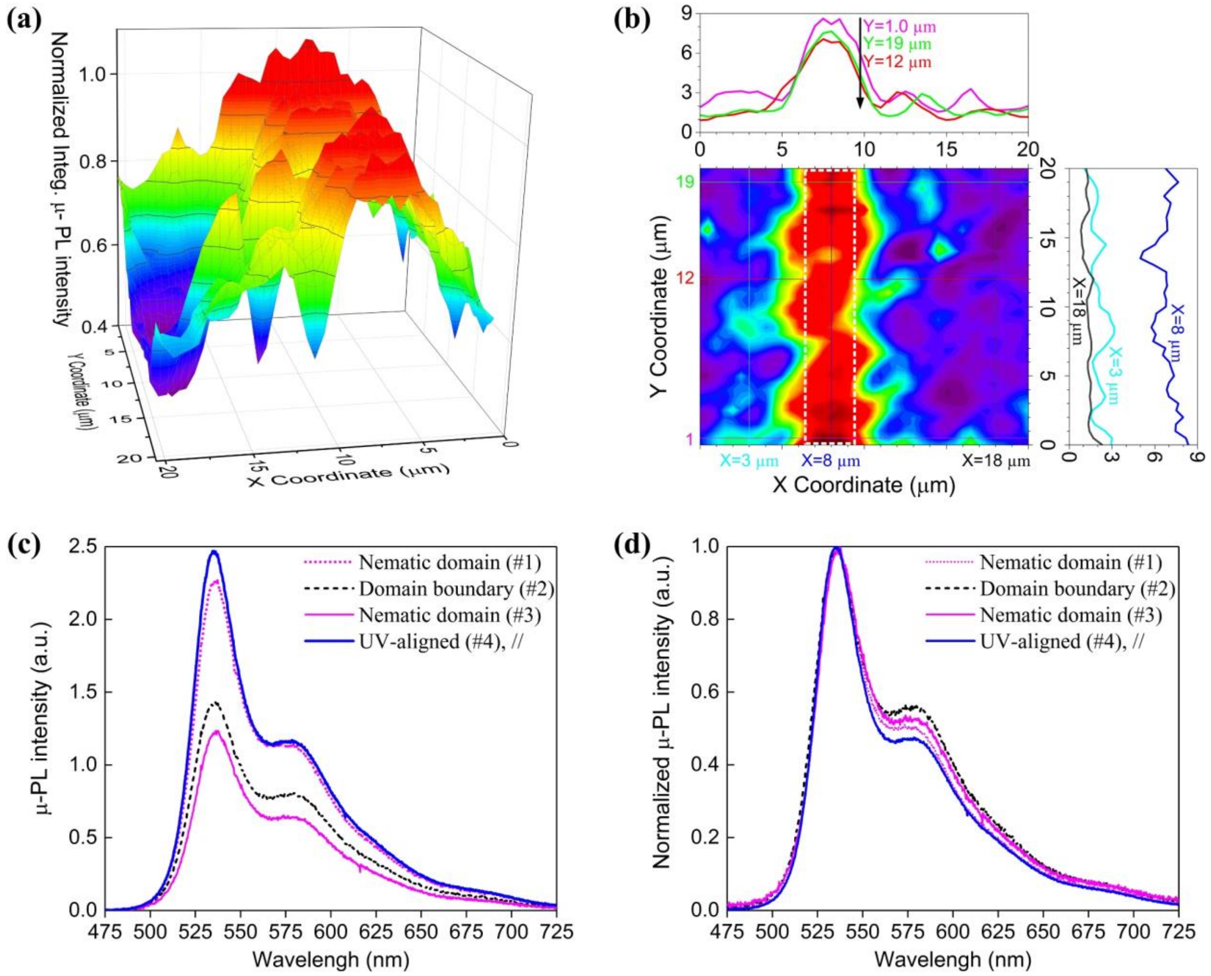

Figure S9. (a) 2-D µ-PL mapping of the integrated $PL_{//}$ spectral intensity for the UV-aligned line (3 µm thick) against the polydomain background. (b) Color-coded contour plot and linecut profiles of the integrated PL anisotropy ratio in the six labelled different lines along the *x*-axis and *y*-axis (i.e., x = 3 µm, 8 µm, and 18 µm; y = 1 µm, 2 µm, and 19 µm) in the colored-coded contour plot. The white dashed box projects the shape and location of the photomask used to pattern the UV alignment of the as-shown 3 µm-thick line in the photoalignment layer. (c) Corresponding $PL_{//}$ spectra and (d) Peak-normalized $PL_{//}$ spectra measured at the four different locations labelled in Figure 4b in the main text. Scanning spot #1 and #3 is located at the trough and peak PL intensity of the nematic domain, respectively, and spot location #2 on the domain boundary and location #4 representing a typical spot in the UV-aligned line.

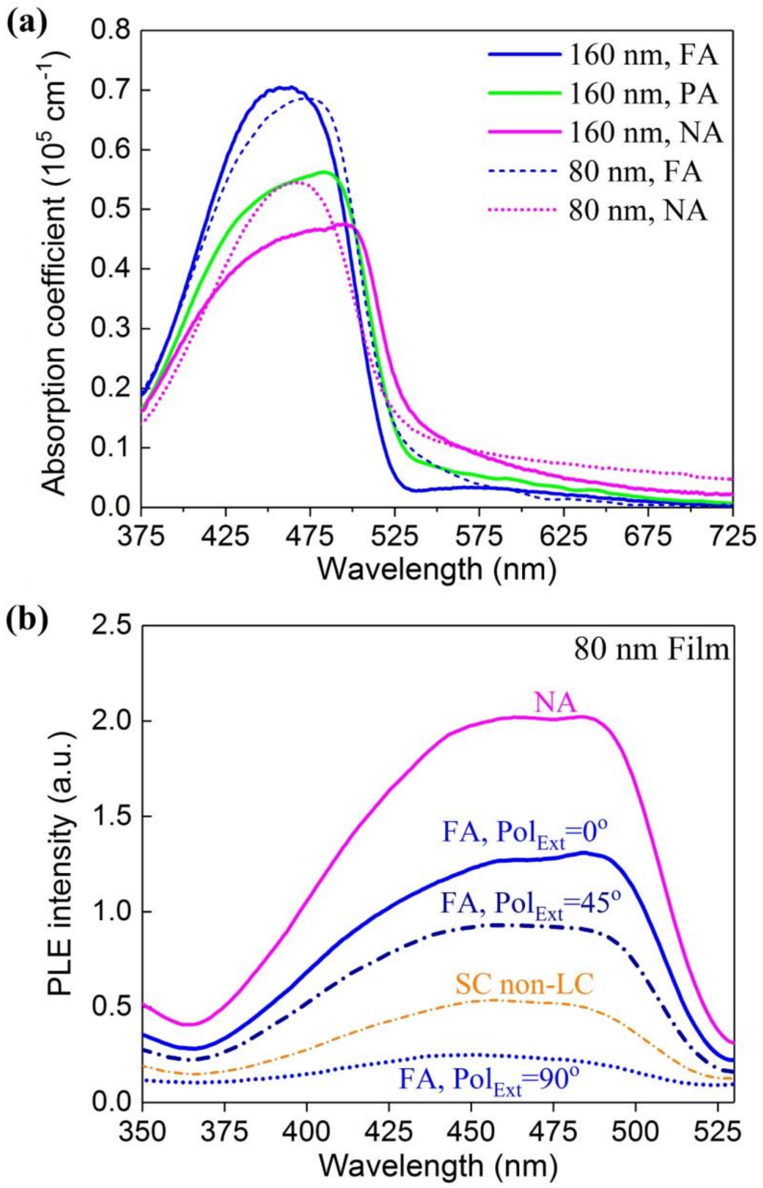

Figure S10. (a) Direct comparison of the absorption coefficient (i.e., the ratio between absorbance and film thickness) spectra in the nonaligned (NA, *Film I*), fully-aligned (FA, *Film II*) and partially-aligned (PA, *Film III*) F8BT nematic glass films with the thickness being 160 nm or 80 nm. (b) Polarized photoexcited (Pol_{Ext} denoting the angle between the excitation polarisation and the chain alignment direction) and non-polarised collected PL excitation (PLE) spectra of the different F8BT films for emission at the 0-0 vibronic PL peak. All measured films in (b) are 80 nm in thickness. We also found that the corresponding PLE spectra for 570 nm emission (not shown here) demonstrate the same trend as these for the 0-0 vibronic PL peak herein.

SECTION III. Domain-size Engineering and Scaling vs F8BT Film Thickness

Physical tuning of the domain boundaries in the nematic polydomain glass films complements the photo-patterning of the chain orientation as to tune the photophysical properties of the F8BT films. A deciding factor for the spatially averaged domain size and, thus, the fraction of domain boundary regions in the self-assembled F8BT nematic polydomains is the thickness of the glass films. **Figure S11** showcases the results of a demonstration of domain engineering via systematically varying the thickness of the *nonaligned* F8BT nematic glass films (*Film I*) and its merit in enhancing the PLQE. The thickness of the nonaligned F8BT nematic glass films was tuned from 40 nm to 480 nm (measured using a Dektak profilometer) by only varying the speed of the spin-coating deposition with the 30 mg/mL F8BT solution in anhydrous toluene. The colours in the crossed polarising optical microscopy (POM) images visualise the phase difference of the nonaligned F8BT glass films due to different thicknesses. Figure S11f demonstrates that the increase in the spatially-averaged domain size with the film thickness in the nonaligned F8BT nematic polydomain films is nearly linear. Similar size scaling trends have been reported for other LCs, e.g., the thickness dependence of the lateral size of domains in Smectic LCs[4] and defect number density in the Schlieren textures of low molar mass nematic LCs.[5]

Given the shape irregularity of the polydomain LC texture observed in the nonaligned F8BT nematic glass films, the size of the nematic domains (w) would scale with the film thickness (D) according to a power law, namely $w \propto D^{H_x/(3-H_y)}$, as a result of a minimization of the energy of the domain bulk against the domain boundary energy.[6,7] Here, H_x and H_y denotes the Hausdorff dimensionality of the irregular F8BT nematic domains along the x-axis and y-axis in the film plane and can be then estimated as $H_x = H_y \approx 1.6$ using the extracted coherence lengths for the nonaligned F8BT nematic film.[8] Taking all together, the scaling of the domain size in the nonaligned F8BT nematic films follows: $w \propto D^{1.14}$, and a fitting result using this power law to the dataset of spatially averaged nematic domain size is illustrated by the black dashed curve in Figure S11f. The minor deviations in fitting the domain size for the nonaligned F8BT nematic films with thickness <80 nm are likely caused by the enhanced surface confinement effect of the reorientation of F8BT chains and altered chain-entanglement state in the nematic mesophase in these ultrathin F8BT films.[9,10] These surface limiting factors were not considered in the proposed simple energy minimization model but may result in the formation of polydomain LC textures in sufficiently thin F8BT nematic glass films.

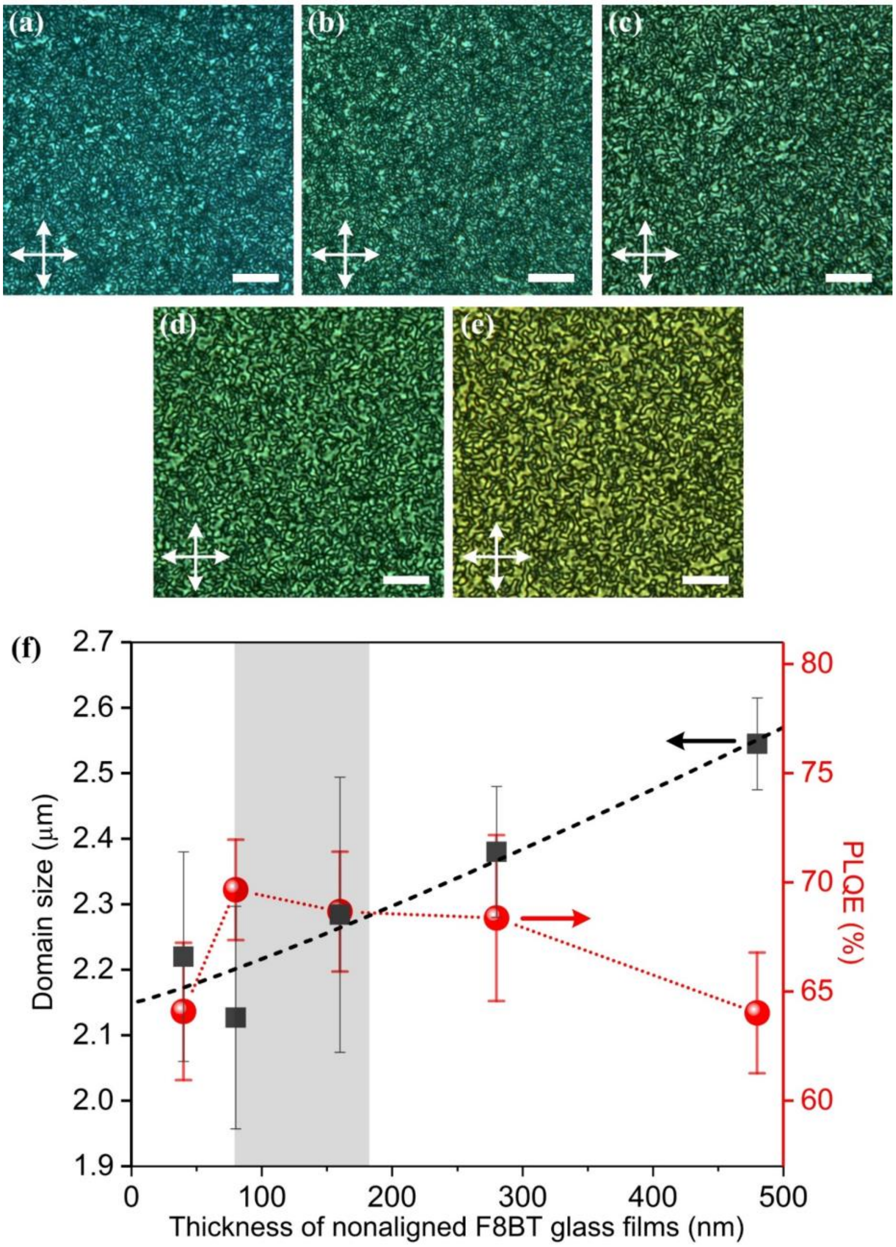

Figure S11. (a - e) Typical POMs demonstrating a polydomain LC texture observed in the quenched nonaligned F8BT nematic films (*Film I*) coated on a quartz substrate with a film thickness of 40 nm (a), 80 nm (b), 160 nm (c), 280 nm (d) and 480 nm (e), viewed between crossed polarisers as indicated by the two crossed white double-headed arrows. The scale bars in (a - e) are 20 μm; the colour change from (a) to (e) corresponds to an increase in retardation in the thicker films. (f) Plots of both the spatially averaged domain size and PLQE as a function of the thickness of the nonaligned nematic F8BT films. The black dashed curve represents the fitting result using the power-law scaling to the dataset for the nematic domain size. The error bars denote a variation in the corresponding data determined from four nonaligned F8BT nematic samples for each film thickness. The shaded rectangle highlights the varying range of the backbone length of the used F8BT.

SECTION IV. Photoalignment of F8BT Nematic Films with Varying Thickness

The F8BT chain-orientation quality and LC domain pattern can be tuned by tailoring the thickness of the SD1-aligned F8BT nematic glass films that were oriented by a UV-aligned (for 5 mins) continuous SD1 layer (deposited from 0.5 mg/ml SD1 solution) before quenching the SD1/F8BT bilayers to form a solid-state glass film. The thickness of the overlying F8BT nematic glass films was engineered from 40 nm to 480 by only varying the speed of the spin-coating deposition with the 30 mg/mL F8BT solution in anhydrous toluene. The thickness dependence of the F8BT film dichroic ratio and the LC textures of these F8BT glass films are shown in **Figure S12a**. It is evident that polymer chain orientation arising from the same UV-aligned SD1 alignment layers is favoured for the F8BT glass films with thicknesses in the range ~100 to 300 nm, in terms of >8 dichroic ratios. The maximum dichroic ratio is 12.3 at a film thickness of ~190 nm, which reaches the theoretical upper limit of dichroic ratio (*DR*) by considering a deviation angle of 20° - 22° between F8BT chain axis and the transition dipole moment. Although a further increase in F8BT film thickness can constantly lower the overall F8BT alignment quality averaged across the whole thickness of the glass films, it reassuringly demonstrates that the best chain orientation occurs for film thicknesses similar to those commonly used in a rich range of device structures.[11-13] This optimised optical dichroism for the photoaligned nematic F8BT in the solid glass nematic state is larger than the DR values reported for the oriented F8BT films by rubbed high-temperature PI alignment layer (*DR* = 8.2)[14] and by surface confinement (*DR* = 7),[15] as well as for the oriented F8BT nanofibers by an electrospinning process (*DR* = 2 -3).[16]

The polymer chains in the mesophase behave in a cooperative manner with the SD1 commanding layer acting to direct their long-range orientational ordering, most desirably into an extended monodomain state. It is expected that the interplay between chain-chain and chain-SD1 interactions facilitates the polymer chain ordering. As for a thicker F8BT film, the aligning effect/force of the optimised SD1 commanding layer will decrease with an increasing distance between the polymer chains and the alignment surface; this tends to magnify the effect of the self-organisation in the nematic phase before quenching. This could explain the emergence of the non-aligned or not-well-aligned regions in the LC textures of the 280 nm-thick Film #4 and 480 nm-thick Film #5 shown in Figure S12b and c. On the other hand, the nematic mesophase polymer chains in a sufficiently thin (e.g., 40 nm - 80 nm in thickness) F8BT glass film may experience a different yet strong surface effect during the SD1-orienting process. This is due to the significantly reduced separation distance between the SD1 alignment surface and the free

surface of the nematic F8BT, which would limit the re-orientation of the polymer chains[17,18] and result in the formation of not-well aligned regions and/or a polydomain texture.

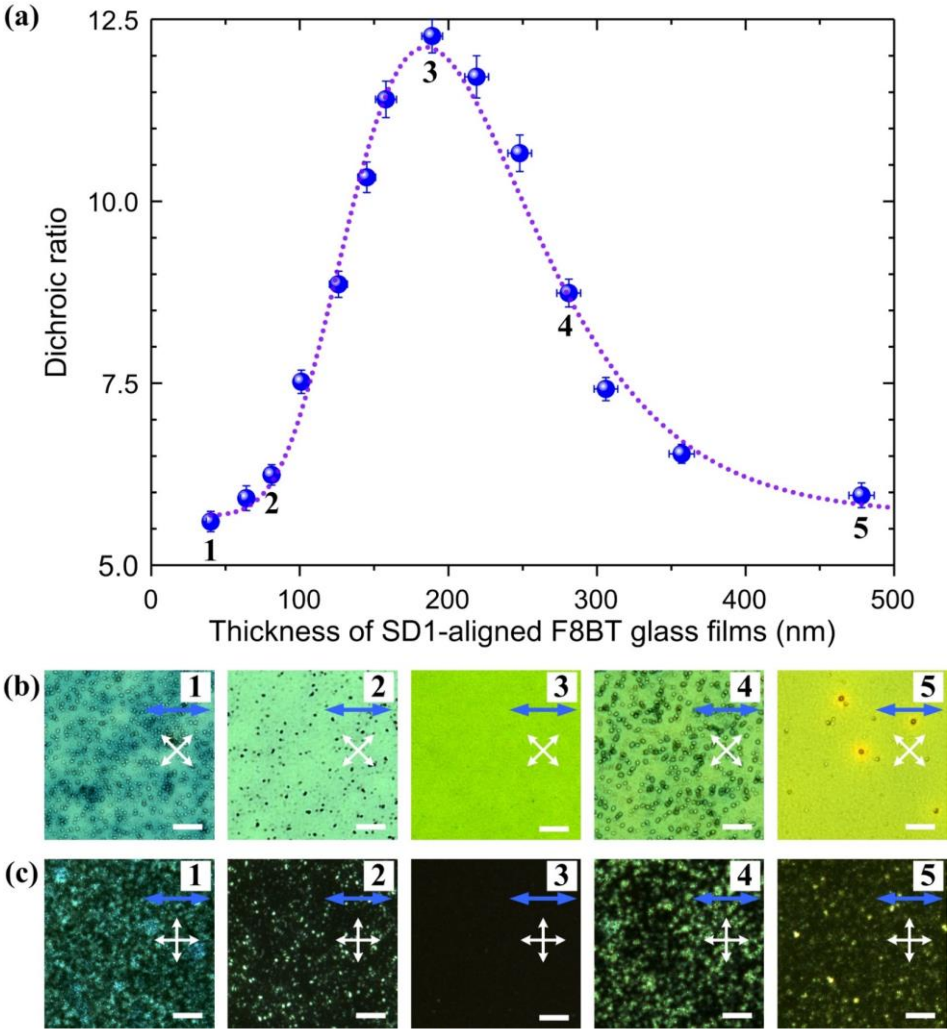

Figure S12. (a) Dichroic ratio in the SD1-aligned (by the UV-illuminated continuous SD1 photoalignment layers) F8BT nematic glass film as a function of F8BT film thickness. The dotted curve is a guide for the eye. (b) Bright-state and (c) Dark-state POM images illustrating the LC textures observed in the SD1-oriented F8BT nematic films with one of the F8BT film thicknesses at the five points labelled in (a), that is, from left to right: 40 nm (1), 80 nm (2), 190 nm (3), 280 nm (4) and 480 nm (5). The horizontal scale bar in each image in (b) and (c) is 20 µm. The oriented F8BT samples (chain orientation denoted by the thick double-headed blue arrows) were placed between a crossed polarised polariser/analyser pair (the crossed thin white arrows). The error bars denote a variation in the corresponding data determined from four samples for each type of F8BT film. The shaded rectangle highlights the varying range of the backbone length of the used F8BT co-polymer. The dark (bright)-state POM images were recorded using the same imaging settings. The colour change from (1) to (5) in (b) corresponds to the increase in retardation in the thicker films.

SECTION V. PL Transients and Lifetime Results

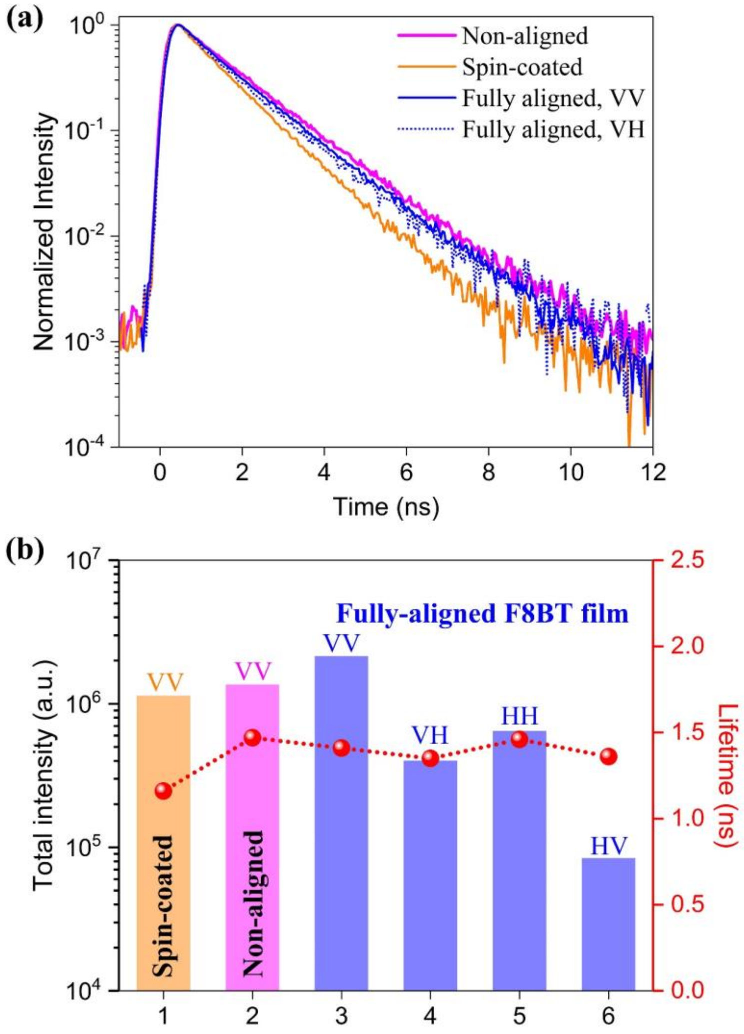

Figure S13. (a) Peak-normalized decay of PL intensity in a fully-aligned 160 nm-thick F8BT nematic monodomain film. (b) The corresponding total PL intensity (bars) and extracted lifetime (stars) from a single-exponential fitting for various 160 nm F8BT films including the spin-coated non-LC, non-aligned nematic, and photoaligned nematic films. The excitation laser for these polarized PL transients remained linearly polarized along the vertical (*V*) direction in the vertical plane. The labels used in (a) - (b) is defined: the first letter (*V* for vertical configuration or $\theta_{Ext} = 0°$ direction; *H* for horizontal or $\theta_{Ext} = 90°$) represents the chain alignment direction in the photoaligned F8BT films, and the second label (*V* or *H*) for the direction of PL collection polarization.

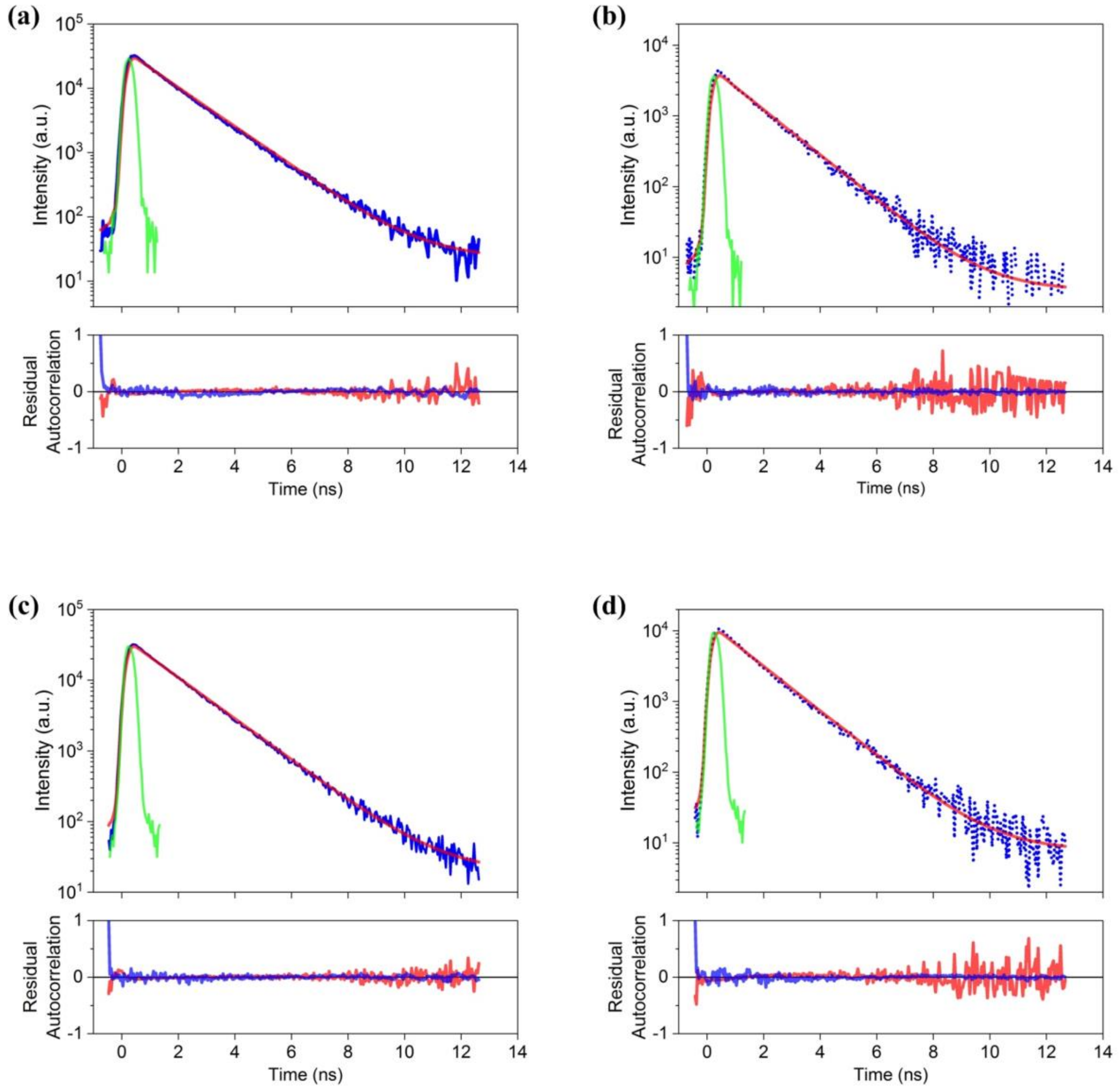

Figure S14. (a) Laser-convolved single exponential fitting of the PL transients recorded from a fully-aligned F8BT nematic film with a thickness of 80 nm (a, b) and 160 nm (c, d). The time-resolved emission was photoexcited by the same parallel polarized laser aligned to the direction of alignment of F8BT chains. (a, c) and (b, d) represent the collection of emission with polarization being aligned parallel or perpendicular to the chain alignment direction in the F8BT film, respectively.

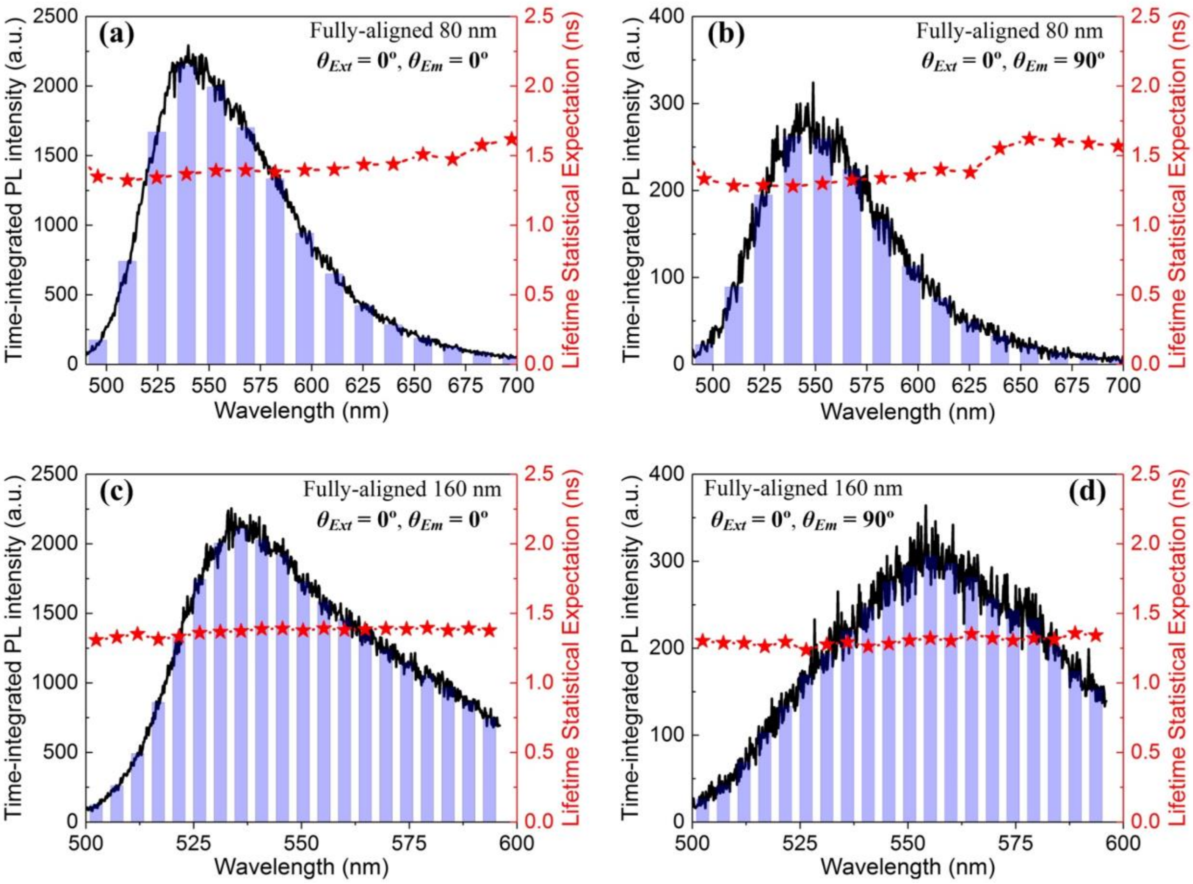

Figure S15. (a) Single-exponential fitting lifetime of the polarized PL transients in the binned spectral ranges for a fully-aligned F8BT nematic film with a thickness of 80 nm (a, b) and 160 nm (c, d). The time-resolved PL signals were photoexcited by the same linearly polarized laser with polarization aligned parallel to the direction of F8BT chain alignment, that is, θ_{Ext} = 0° for all cases. (a, c) and (b, d) represent collection of the emission for light aligned parallel (θ_{Em} = 0°) or perpendicular (θ_{Em} = 90°) to the chain direction, respectively.

SI References

[1] C. Nicklin, J. Martinez-Hardigree, A. Warne, S. Green, M. Burt, J. Naylor, A. Dorman, D. Wicks, S. Din, M. Riede *Rev. Sci. Instrum.* **2017**, 88, 103901.

[2] J. Filik, A. W. Ashton, P. C. Y. Chang, P. A. Chater, S. J. Day, M. Drakopoulos, M. W. Gerring, M. L. Hart, O. V. Magdysyuk, S. Michalik, A. Smith, C. C. Tang, N. J. Terrill, M. T. Wharmby, H. Wilhelm *J. Appl. Cryst.* **2017**, 50, 959.

[3] J. C. de Mello, H. Felix Wittmann, R. H. Friend *Adv. Mater.* **1997**, 9, 230.

[4] I. Gryn, E. Lacaze, R. Bartolino, B. Zappone *Adv. Funct. Mater.* **2015**, 25, 142.

[5] T. Ohzono, K. Katoh, C. Wang, A. Fukazawa, S. Yamaguchi, J. Fukuda *Sci. Rep.* **2017**, 7, 16814 .

[6] G. Catalan, H. Béa, S. Fusil, M. Bibes, P. Paruch, A. Barthélémy, J. F. Scott *Phys. Rev. Lett.* **2008**, 100, 027602.

[7] G. Catalan, J. Seidel, R. Rames, J. F. Scott *Rev. Mod. Phys.* **2012**, 84, 119.

[8] E. B. Burlakova, A. E. Shilov, S. D. Varfolomeev, G. E. Zaikov (eds.), *Chemical and Biological Kinetics*. New Horizons. Vol. 1. *Chemical Kinetics*, VSP Int. Publ., Leiden-Boston **2005**.

[9] N. Lee, D. Diddens, H. Meyer, A. Johner *Phys. Rev. Lett.* **2017**, 118, 067802.

[10] A. Milchev, K. Binder *Phys. Rev. Lett.* **2019**, 123, 128003.

[11] R. Xia, C. Cheung, A. Ruseckas, D. Amarasinghe, I. D. W. Samuel, D. D. C. Bradley *Adv. Mater.* **2007**, 19, 4054.

[12] C. I. Wilkinson, D. G. Lidzey, L. C. Palilis, R. B. Fletcher, S. J. Martin, X. Wang, D. D. C. Bradley *Appl. Phys. Lett.* **2001**, 79, 171.

[13] T. Virgili, D. G. Lidzey, M. Grell, D. D. C. Bradley, S. Stagira, M. Zavelani-Rossi, S. De Silvestri *Appl. Phys. Lett.* **2002**, 80, 4088.

[14] R. Xia, M. Campoy-Quiles, G. Heliotis, P. Stavrinou, K. S. Whitehead, D. D. C. Bradley *Synth. Met.* **2005**, 15, 274.

[15] Z. Zheng, K.-H. Yim, M. S. M. Saifullah, M. E. Welland, R. H. Friend, J.-S. Kim, W. T. S. Huck *Nano Lett.* **2007**, 7, 4, 987.

[16] S. Pagliara, A. Camposeo, E. Mele, L. Persano, R. Cingolani, D. Pisignano *Nanotechnology* **2010**, 21, 215304.

[17] N. Lee, D. Diddens, H. Meyer, A. Johner *Phys. Rev. Lett.* **2017**, 118, 067802.

[18] A. Milchev, K. Binder *Phys. Rev. Lett.* **2019**, 123, 128003.
