# Supplementary material for: Fine‐Tuning the Microstructure and Photophysical Characteristics of Fluorescent Conjugated Copolymers Using Photoalignment and Liquid‐Crystal Ordering
Source: Adv Sci (Weinh). 2024 Aug 29;11(41):2407117. doi: 10.1002/advs.202407117 (PMC11538637; doi:10.1002/advs.202407117)
Supplement: Supplementary file 3 — Supplemental Movie [file ADVS-11-2407117-s001.docx]

Supplementary Information

**SI Movie and Description Text**

This Supporting Information Movie demonstrates a continuous transition between the bright state and dark state in a set of photoaligned F8BT lines [line width: 250 μm (×1), 3.0 μm (×4), 5 μm (×4), 10 μm (×3); line separation: 100 μm] against the self-assembled nematic micro-domains in the same F8BT glass film sample, observed in a high-resolution crossed-polarized optical microscopy when rotating the patterned F8BT film in the plane of the substrate. The as-shown high-quality chain alignment in the photoaligned lines was created via photo-masked UV-alignment of the continuous photoalignment layer; then the photoaligned pattern was transferred into the overlaying F8BT film (160 nm thickness) in the process of thermotropic alignment arising from the long-range liquid-crystalline ordering of polymer chains.
